# Creation of a Pre-biopsy Clinical Risk Score for Major Bleeding After Kidney Biopsy

**DOI:** 10.1016/j.ekir.2026.107118

**Published:** 2026-10-01

**Authors:** Rena Suzukawa, Daiei Takahashi, Takanori Hasegawa, Shunsuke Inaba, Ken Ikenouchi, Yuta Nakano, Yurina Yataka, Mizuki Watada, Motoki Hoshino, Wakana Shoda, Tamaki Kuyama, Eisei Sohara, Kiyohide Fushimi, Shinichi Uchida, Shintaro Mandai

**Affiliations:** 1 Department of Nephrology, Graduate School of Medical and Dental Sciences, Institute of Science Tokyo, Tokyo, Japan; 2 Department of Nephrology, Musashino Red Cross Hospital, Musashino, Tokyo, Japan; 3 M&D Data Science Centre, Institute of Integrated Research, Institute of Science Tokyo, Tokyo, Japan; 4 Department of Population Health Sciences, University of Bristol, Bristol, Bristol, UK

**Keywords:** administrative database, major bleeding, percutaneous kidney biopsy, prebiopsy clinical diagnosis, prediction scoring, risk stratification

## Abstract

**Introduction:**

Risk-based tools to guide care after percutaneous kidney biopsy (PKB) remain limited. We developed a simple preadmission risk score to predict post-PKB bleeding and support same-day discharge decisions, leveraging Japan’s routine inpatient observation practice.

**Methods:**

This retrospective study included 78,817 adults undergoing native PKB, using a nationwide Japanese inpatient database (2016–2021). Risk factors for early hemostatic intervention, defined as blood transfusion or surgical or interventional hemostasis within 6 days after PKB, were evaluated using logistic and L2-penalized multinomial logistic regressions. The fitted coefficients for day 0 were discretized to develop the Kidney biopsy Bleeding Index for Triage (K-BIT), using readily obtainable prebiopsy clinical variables. The scoring was internally validated using a held-out test cohort from the same dataset.

**Results:**

The overall bleeding rate was 1.46%. Compared with chronic glomerulonephritis, bleeding risk was higher in acute kidney injury (odds ratio [OR], 10.9; 95% confidence interval [CI], 8.55–13.9) and rapidly progressive glomerulonephritis (OR, 6.67; 95% CI 5.48–8.12). Increasing age and lower body mass index (BMI) were independently associated with higher bleeding risk. Age, prebiopsy diagnostic risk tier, BMI, and Charlson Comorbidity Index (CCI) were selected for score construction (score range, 0–9). In the validation cohort, patients with scores ≤ 2 (55.4%) had a bleeding risk of 0.37%. Simulation analysis suggested that restricting early discharge to this low-risk group could reduce hospitalization by an estimated 332 hospital days per 100 patients while maintaining low postdischarge bleeding risk.

**Conclusion:**

Prebiopsy diagnosis, age, BMI, and comorbidities were key predictors of postbiopsy bleeding. K-BIT may support risk stratification and outpatient kidney biopsy pathways.

## Graphical abstract

**Figure:**
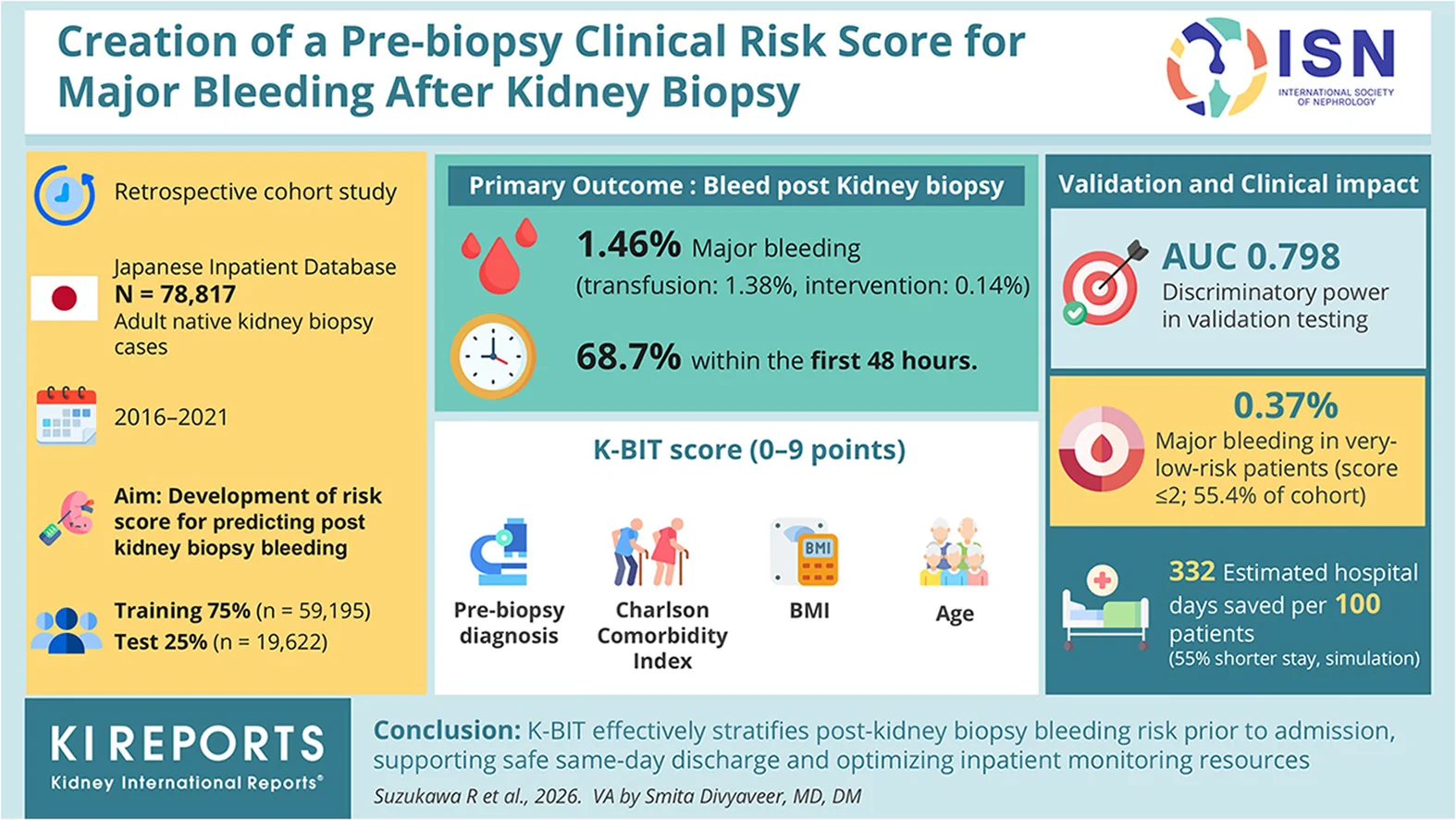

PKB is an indispensable diagnostic tool in nephrology. The histopathologic information obtained through this procedure facilitates diagnosis and disease classification, guides therapeutic decisions, and contributes to prognostic assessment.^1^ Despite its diagnostic utility, PKB carries a risk of bleeding complications, ranging from minor events to serious major bleeding requiring blood transfusion, endovascular therapy, or surgical intervention, and rarely resulting in death.^1^, ^2^, ^3^, ^4^, ^5^, ^6^

Postbiopsy management after PKB remains heterogeneous across centers, even within a single national health care system.^7^, ^8^ International practice has increasingly shifted toward a risk-based approach, including same-day discharge for selected low-risk patients after several hours of observation. The Kidney Health Australia–Caring for Australasians with Renal Impairment guideline endorses 6–8 hours of observation followed by same-day discharge for low-risk patients, whereas advising longer inpatient monitoring, typically up to 24 hours, for those at greater risk of postbiopsy complications.^9^ Although the optimal observation period remains controversial,^10^ several studies have evaluated short-stay or outpatient kidney biopsy protocols.^11^, ^12^, ^13^, ^14^ Against this background, routine hospitalization after PKB remains common in Japan, with a median stay of 5–6 days.^8^ This practice offers a unique opportunity to capture the timing of complications after biopsy and to evaluate and identify risk factors for both immediate complications occurring within the first day after PKB and less predictable complications with later onset.

Several scoring systems to predict the risk of complications after PKB have been developed and validated,^15^, ^16^, ^17^, ^18^, ^19^, ^20^, ^21^, ^22^ and many use clinically relevant prebiopsy information such as hemoglobin, platelet count, kidney size, comorbidities, and kidney function. These models have advanced individualized risk assessment and provided important tools for procedural decision-making. However, most existing tools were not specifically designed to support preadmission triage decisions. Although some previous risk models, including the nationwide French score developed by Halimi *et al.*,^21^ incorporated diagnosis-related categories, evidence remains limited regarding how preadmission clinical information available during outpatient evaluation can be translated into postbiopsy care pathways and discharge planning. Some conditions, such as systemic lupus erythematosus^23^ and amyloidosis,^24^ have been specifically investigated in relation to the risk of postbiopsy complications. Nonetheless, PKB is performed for a wide range of clinical conditions, including chronic glomerulonephritis, nephrotic syndrome, autoimmune diseases, and acute kidney injury, each of which may carry different baseline risks of bleeding. Furthermore, variability in reported complication rates, partly due to differences in outcome definitions and single- or multicenter cohorts,^4^, ^6^ makes the validation of scoring systems less straightforward.

To address these limitations and improve applicability, we conducted a nationwide study using the Japanese administrative claims inpatient database, a reliable source for clinical research^25^, ^26^ that captures more than half of all hospital admissions in Japan. The aim of this study was to identify preadmission clinical variables associated with postbiopsy bleeding and to develop a simple, clinically interpretable risk score to help identify low-risk patients who could be considered for same-day discharge after PKB, leveraging Japan’s routine practice of inpatient observation after PKB. To our knowledge, this scoring system was derived from one of the largest cohorts of patients undergoing PKB, enabling robust split-sample validation and ensuring sufficient outcome events within each diagnostic category and stratified patient subgroup.

## Methods

### Study Design and Data Source

This is a retrospective cohort study that analyzed data from the Diagnosis Procedure Combination inpatient database (2016–2021), a Japanese nationwide inpatient administrative database covering acute-care hospitalizations.^25^, ^26^, ^27^ It contains deidentified, admission-level information submitted by participating hospitals, including demographic characteristics, dates of admission and discharge, *International Classification of Diseases and Related Health Problems, 10*^*th*^ *Revision (ICD-10)*^28^ diagnosis codes, recorded comorbidities, procedures, prescribed drugs, discharge disposition, length of stay, and in-hospital outcomes. This study was approved by the ethics committee of the Institute of Science Tokyo (approval number M2000-788). Informed consent was waived because this retrospective study used anonymized data. The study was conducted in accordance with the Declaration of Helsinki and was reported in accordance with the STROBE guidelines for observational studies and the TRIPOD guidelines for clinical prediction model studies. Diagnosis Procedure Combination data cannot be shared; methodological details are available upon reasonable request.

### Eligibility Criteria

We included patients aged 18 years or older undergoing native PKB, in whom the primary diagnoses, the reason for admission, or the disease associated with the highest medical care costs corresponded to renal diseases (Figure 1 and Supplementary Table S1). Exclusion criteria were as follows: aged < 18 years, PKB performed after hospital day 7, kidney transplant recipients, patients with tumor-related kidney diseases (*ICD-10* codes: C64, C65, and D41.0), patients with missing height, weight, or body mass index (BMI) data, and extreme BMI (< 15 or ≥ 50 kg/m^2^). After applying these criteria, 78,817 patients were included in the final analysis cohort. Patients were followed from the date of kidney biopsy until discharge, transfer, or in-hospital death.^29^

**Figure 1:**
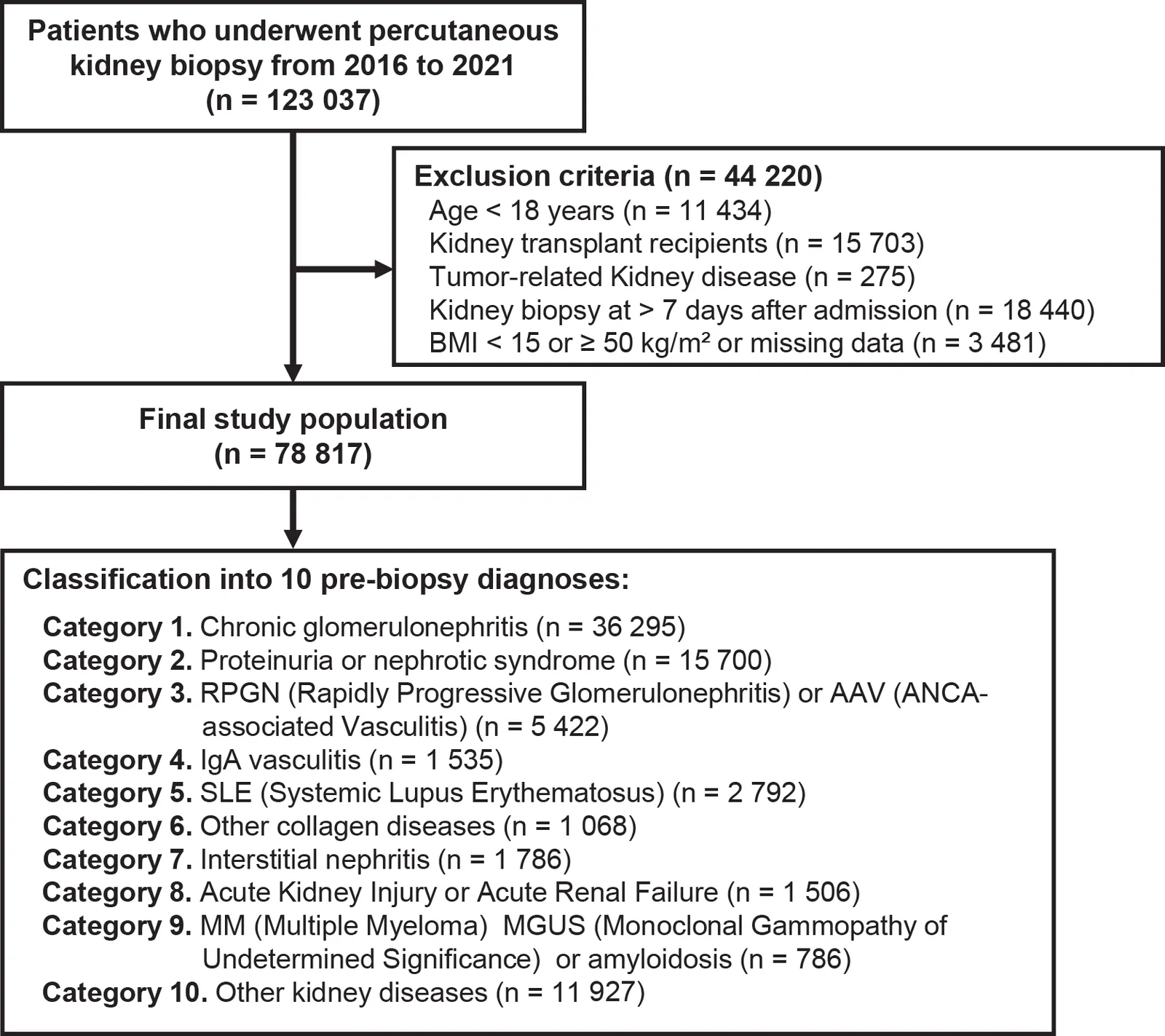
Flow diagram of patient selection. In total, 78,817 patients were included in the final analytic cohort. Patients were categorized into 10 clinical diagnostic groups based on admission diagnoses. AAV, antineutrophil cytoplasmic antibody–associated vasculitis; BMI, body mass index; MGUS, monoclonal gammopathy of undetermined significance; MM, multiple myeloma; RPGN, rapidly progressive glomerulonephritis; SLE, systemic lupus erythematosus.

### Data Collection and Patient Stratification

We collected data on age, sex, BMI, admission and discharge status, medications prescribed, comorbidities, and CCI,^30^ which is updated for risk adjustment.^29^, ^31^ Patients were stratified into 5 age groups based on their age (18–49, 50–59, 60–69, 70–79, and ≥ 80 years),^32^ and grouped into 4 BMI categories (< 18.5, 18.5–< 22.0, 22.0–< 25.0, and ≥ 25.0 kg/m^2^). The primary prebiopsy diagnosis was categorized into 10 clinically relevant groups based on *ICD-10* codes (Figure 1 and Supplementary Table S1). Given that hemostatic agents are administered immediately before kidney biopsy in routine clinical practice in Japan, hemostatic medication use on the day of biopsy was identified using medication claims and categorized as follows: no agent used, tranexamic acid alone, carbazochrome sulfonate alone, and combination use. Of note, laboratory and imaging data were unavailable in our reimbursement claims-based database. Because guidelines in Japan recommend withholding antithrombotic agents for approximately 1 week before kidney biopsy,^33^ use of these agents was not included as a predictor in the present model.

### Outcomes

The primary outcome was major bleeding, defined as the first occurrence of either blood transfusion or surgical or interventional hemostasis within the first 6 days after PKB. Surgical or interventional hemostasis included blood vessel ligation, endovascular intervention including vascular embolization, and nephrectomy. Death alone was not considered a primary outcome event because the specific cause of death could not be determined and was likely attributable to various underlying factors.

### Statistical Analysis

Descriptive statistics were expressed as numerical values (%) and medians with interquartile ranges (IQR). Proportions and their 95% confidence intervals were calculated using the Wald method based on the normal approximation. We used logistic regression to estimate odds ratios (ORs) and 95% confidence intervals (CIs) for the risk of major bleeding, adjusting for year (2016–2017, 2018–2019, and 2020–2021) of biopsy, age, sex, BMI, CCI, diabetes, cardiovascular disease, prebiopsy clinical diagnostic category, and use of hemostatic agents. Candidate predictors were selected with reference to previous publications based on clinical relevance and availability.^15^, ^16^, ^17^, ^18^, ^19^, ^20^, ^21^, ^22^, ^34^, ^35^, ^36^, ^37^ Sensitivity analyses were also conducted that limited the outcome to blood transfusion alone or to an initial transfusion dose of ≥ 4 units. The workflow for model development and score generation is depicted in Supplementary Figure S1.

For model development, the outcome was categorized into 8 mutually exclusive postoperative bleeding-timing categories: nonoccurrence, and the specific day of the first major bleeding event (day 0–6) and was arranged *a priori* according to clinical timing. To model the risk of major bleeding and its timing, we employed L2-penalized multivariable multinomial logistic regression with the variables selected above, excluding diabetes and cardiovascular disease and additionally including hospital size (10-category annual dialysis inpatient volume), using training data from 4 regions in Japan (Kanto: 23,357 [29.63%], Kansai: 13,705 [17.39%], Chubu: 13,809 [17.52%], Hokkaido/Tohoku: 8324 [10.56%]; total n = 59,195). Details of the modeling design and visualization are provided in the Supplementary Methods.

To construct the K-BIT scoring system, we examined the estimated coefficients for all outcome levels. Variables with the strongest absolute associations and clinical interpretability on day 0 were selected, because this time point had the highest event frequency and best captured acute bleeding risk relevant to discharge decisions. In contrast, late-onset bleeding events were less frequent and considered more difficult to predict, likely because their causes and contributing factors vary among patients. Variables with minimal contributions or unclear clinical relevance were excluded from the model. To generate the point values, the selected coefficients were divided by 0.3 and then discretized into integers. This scaling factor was chosen to preserve the relative weight of each variable while yielding a clinically usable score range.

Internal validation was performed using an independent test cohort derived from the same dataset covering 2 Japanese regions (Chugoku/Shikoku: 9007 [11.43%], Kyushu/Okinawa: 10,615 [13.47%]; total *n* = 19,622; Supplementary Figure S1). For comparison with conventional binary outcome evaluation, we additionally performed a binary receiver operating characteristic analysis. The multiple bleeding-timing categories were combined into a binary outcome: any bleeding event versus no bleeding. The predicted probability of any bleeding event was calculated by summing the predicted probabilities across all bleeding categories. Model discrimination for any bleeding event was assessed using the area under the curve (AUC, with 95% confidence intervals calculated by DeLong’s method). Analyses were conducted using JMP Pro 17.0 (SAS Institute Inc., Cary, NC, USA), Stata version 18.0 (Stata Corp, College Station, TX, USA), and R version 4.5.0 (R Foundation for Statistical Computing, Vienna, Austria). A 2-sided *P* value < 0.05 was considered statistically significant.

## Results

### Patient Characteristics and Classification

In total, 123,037 PKB cases in Japan were initially identified between 2016 and 2021 (Figure 1). Of these, 78,817 were ultimately included in the analysis. Patients were grouped into 10 diagnostic categories. The median hospital stay was 6 days and the mean was 13 days. During follow-up, 1102 patients (1.4%) were transferred to another hospital or clinic and 423 patients (0.5%) died before discharge, as identified from discharge information.

Patient characteristics (stratified by age) are summarized in Table 1. The median age was 55 years (IQR, 40–69 years), and 47.3% of the cohort was female. The median BMI was 23.07 kg/m^2^, with the highest median observed in patients aged 50–59 years (23.90 kg/m^2^) and the lowest in those aged 18–49 years (22.55 kg/m^2^). Underweight status (BMI < 18.5 kg/m^2^) was more frequently observed in both the youngest (9.9%) and oldest (8.8%) age groups. Most of the cohort (59.5%) had a normal BMI (18.5–< 25.0 kg/m^2^). Although 61.4% of the cohort had a CCI of 0, a higher comorbidity burden (CCI ≥ 3) was more prevalent in older patients. Diabetes was present in 17.4% of patients, including diabetes without chronic complications (11.5%) and diabetes with chronic complications (5.9%). Hemostatic agents were used in 70.9% of patients, most commonly as a combination of tranexamic acid and carbazochrome sulfonate (49.9%), followed by carbazochrome sulfonate alone (16.3%) and tranexamic acid alone (4.7%).

**Table 1:** Characteristics of patients who underwent a kidney biopsy in Japan in 2016–2021

Variables	Whole group (*n* = 78,817)	Age 18–49 (*n* = 32,070)	Age 50–59 (*n* = 12,465)	Age 60–69 (*n* = 15,810)	Age 70–79 (*n* = 14,593)	Age ≥ 80 (*n* = 3879)
Median age (IQR), yr	55 (40–69)	37 (28–44)	54 (52–57)	65 (62–67)	74 (72–76)	82 (81–84)
Female sex, *n* (%)	37,306 (47.33%)	16,979 (52.94%)	5696 (45.70%)	6643 (42.02%)	6151 (42.15%)	1837 (47.36%)
Median BMI (IQR), kg/m^2^	23.07 (20.62–25.97)	22.55 (20.16–25.81)	23.90 (21.29–26.93)	23.56 (21.17–26.14)	23.07 (20.78–25.51)	22.76 (20.48–25.04)
BMI category, kg/m^2^, *n* (%)						
<18.5	6525 (8.28%)	3175 (9.90%)	758 (6.08%)	1058 (6.69%)	1191 (8.16%)	343 (8.84%)
18.5 – < 22.0	23,958 (30.40%)	11,109 (34.64%)	3136 (25.16%)	4169 (26.37%)	4308 (29.52%)	1236 (31.86%)
22.0 – < 25.0	22,960 (29.13%)	8133 (25.36%)	3569 (28.63%)	5108 (32.31%)	4834 (33.13%)	1316 (33.93%)
≥ 25.0	25,374 (32.19%)	9653 (30.10%)	5002 (40.13%)	5475 (34.63%)	4260 (29.19%)	984 (25.37%)
Charlson comorbidity, *n* (%)						
Myocardial infarction	246 (0.31%)	20 (0.06%)	31 (0.25%)	74 (0.47%)	103 (0.71%)	18 (0.46%)
Congestive heart failure	2966 (3.76%)	739 (2.30%)	423 (3.39%)	654 (4.14%)	808 (5.54%)	342 (8.82%)
Peripheral vascular disease	585 (0.74%)	94 (0.29%)	63 (0.51%)	144 (0.91%)	205 (1.40%)	79 (2.04%)
Cerebrovascular disease	1167 (1.48%)	124 (0.39%)	133 (1.07%)	304 (1.92%)	463 (3.17%)	143 (3.69%)
Dementia	186 (0.24%)	4 (0.01%)	2 (0.02%)	18 (0.11%)	74 (0.51%)	88 (2.27%)
Chronic pulmonary disease	2045 (2.59%)	480 (1.50%)	241 (1.93%)	486 (3.07%)	651 (4.46%)	187 (4.82%)
Rheumatologic disease	5177 (6.57%)	2295 (7.16%)	795 (6.38%)	958 (6.06%)	918 (6.29%)	211 (5.44%)
Peptic ulcer disease	2310 (2.93%)	853 (2.66%)	326 (2.62%)	473 (2.99%)	502 (3.44%)	156 (4.02%)
Mild liver disease	2571 (3.26%)	699 (2.18%)	487 (3.91%)	683 (4.32%)	576 (3.95%)	126 (3.25%)
Diabetes without chronic complications	9065 (11.50%)	1841 (5.74%)	1475 (11.83%)	2494 (15.77%)	2616 (17.93%)	639 (16.47%)
Diabetes with chronic complications	4625 (5.87%)	990 (3.09%)	907 (7.28%)	1237 (7.82%)	1204 (8.25%)	287 (7.40%)
Hemiplegia or paraplegia	20 (0.03%)	4 (0.01%)	2 (0.02%)	4 (0.03%)	9 (0.06%)	1 (0.03%)
Renal disease	13,873 (17.60%)	4593 (14.32%)	2421 (19.42%)	3170 (20.05%)	2903 (19.89%)	786 (20.26%)
Any malignancy, including leukemia and lymphoma	4827 (6.12%)	873 (2.72%)	685 (5.50%)	1237 (7.82%)	1531 (10.49%)	501 (12.92%)
Moderate or severe liver disease	52 (0.07%)	9 (0.03%)	10 (0.08%)	19 (0.12%)	11 (0.08%)	3 (0.08%)
Metastatic solid tumor	433 (0.55%)	32 (0.10%)	58 (0.47%)	139 (0.88%)	151 (1.03%)	53 (1.37%)
AIDS/HIV	69 (0.09%)	42 (0.13%)	16 (0.13%)	7 (0.04%)	3 (0.02%)	1 (0.03%)
Charlson Comorbidity Index, *n* (%)						
0	48,428 (61.44%)	22,699 (70.69%)	7454 (59.80%)	8852 (55.99%)	7588 (52.00%)	1865 (48.08%)
1–2	26,073 (33.08%)	8554 (26.67%)	4366 (35.03%)	5812 (36.76%)	5760 (39.47%)	1581 (40.76%)
≥3	4316 (5.48%)	847 (2.64%)	645 (5.17%)	1146 (7.25%)	1245 (8.53%)	433 (11.16%)
Haemostatic agent use, *n* (%)						
None	22,942 (29.11%)	9077 (28.30%)	3724 (29.88%)	4805 (30.39%)	4159 (28.50%)	1177 (30.34%)
Tranexamic acid	3677 (4.66%)	1536 (4.79%)	592 (4.75%)	705 (4.46%)	690 (4.73%)	154 (3.97%)
Carbazochrome sulfonate	12,848 (16.30%)	5593 (17.44%)	1977 (15.86%)	2397 (15.16%)	2291 (15.70%)	590 (15.21%)
Combination therapy	39,350 (49.93%)	15,864 (49.47%)	6172 (49.51%)	7903 (49.99%)	7453 (51.07%)	1958 (50.48%)
Clinical diagnostic groups, *n* (%)						
Category 1: CGN	36,295 (46.05%)	19,077 (59.49%)	6190 (49.66%)	6350 (40.16%)	4061 (27.83%)	617 (15.91%)
Category 2: proteinuria, NS	15,700 (19.92%)	4347 (13.55%)	2052 (16.46%)	3485 (22.04%)	4407 (30.20%)	1409 (36.32%)
Category 3: RPGN, AAV	5422 (6.88%)	620 (1.93%)	587 (4.71%)	1334 (8.44%)	2135 (14.63%)	746 (19.23%)
Category 4: IgAV	1535 (1.95%)	825 (2.57%)	177 (1.42%)	265 (1.68%)	209 (1.43%)	59 (1.52%)
Category 5: SLE	2792 (3.54%)	1899 (5.92%)	422 (3.39%)	292 (1.85%)	155 (1.06%)	24 (0.62%)
Category 6: other collagen diseases	1068 (1.36%)	152 (0.47%)	143 (1.15%)	297 (1.88%)	372 (2.55%)	104 (2.68%)
Category 7: interstitial nephritis	1786 (2.27%)	556 (1.73%)	298 (2.39%)	421 (2.66%)	416 (2.85%)	95 (2.45%)
Category 8: AKI, ARF	1506 (1.91%)	337 (1.05%)	207 (1.66%)	356 (2.25%)	433 (2.97%)	173 (4.46%)
Category 9: MM, MGUS, amyloidosis	786 (1.00%)	71 (0.22%)	112 (0.90%)	217 (1.37%)	286 (1.96%)	100 (2.58%)
Category 10: other kidney diseases	11,927 (15.13%)	4186 (13.05%)	2277 (18.27%)	2793 (17.67%)	2119 (14.52%)	552 (14.23%)
Region, *n* (%)						
Hokkaido and Tohoku	8324 (10.56%)	3005 (9.37%)	1395 (11.19%)	1953 (12.35%)	1595 (10.93%)	376 (9.69%)
Kanto	23,357 (29.63%)	10,092 (31.47%)	3788 (30.39%)	4315 (27.29%)	4120 (28.23%)	1042 (26.86%)
Chubu	13,809 (17.52%)	5788 (18.05%)	2186 (17.54%)	2651 (16.77%)	2519 (17.26%)	665 (17.14%)
Kansai	13,705 (17.39%)	5496 (17.14%)	2107 (16.90%)	2574 (16.28%)	2724 (18.67%)	804 (20.73%)
Chugoku and Shikoku	9007 (11.43%)	3494 (10.89%)	1320 (10.59%)	1852 (11.71%)	1807 (12.38%)	534 (13.77%)
Kyushu and Okinawa	10,615 (13.47%)	4195 (13.08%)	1669 (13.39%)	2465 (15.59%)	1828 (12.53%)	458 (11.81%)

AAV, antineutrophil cytoplasmic antibody–associated vasculitis; AKI, acute kidney injury; ARF, acute renal failure; BMI, body mass index; CGN, chronic glomerulonephritis; IgAV, IgA vasculitis; IQR, interquartile range; MGUS, monoclonal gammopathy of undetermined significance; MM, multiple myeloma; NS, nephrotic syndrome; RPGN, rapidly progressive glomerulonephritis; SLE, systemic lupus erythematosus.

Data presented as *n* (%) or median (IQR).

### Incidence and Timing of Major Bleeding Events

Within 6 days of biopsy, 1091 patients (1.38%) underwent blood transfusion and 109 (0.14%) required surgical or endovascular intervention. Overall, major bleeding events, defined as either blood transfusion or surgical or endovascular intervention, occurred in 1147 patients (1.46%): 0.61% (95% CI, 0.53–0.70) in patients aged 18–49 years, 1.17% (1.00–1.38) in those aged 50–59 years, 1.47% (1.30–1.67) in those aged 60–69 years, 2.76% (2.51–3.04) in those aged 70–79 years, and 4.38% (3.78–5.07) in patients aged ≥ 80 years (Figure 2a).

**Figure 2:**
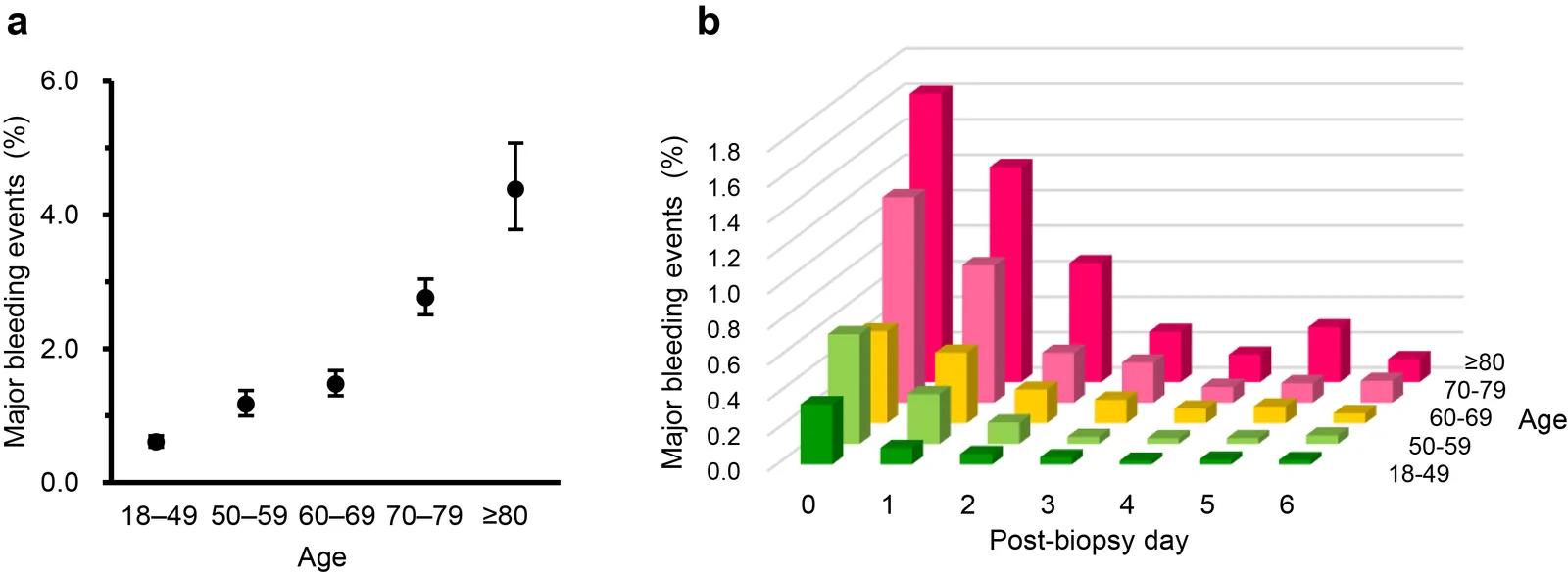
Incidence and timing of major bleeding events after percutaneous kidney biopsy. (a) Incidence of major bleeding events within the first 6 days after percutaneous kidney biopsy, stratified by age group (18–49, 50–59, 60–69, 70–79, and ≥80 years). (b) Distribution of the timing of major bleeding events according to postbiopsy day and age group. The z-axis represents the percentage of events within each age category. Most events occurred on the day of (day 0) and the day after (day 1) the biopsy. Green, 18–49 years; light green, 50–59 years; yellow, 60–69 years; pink, 70–79 years; red, ≥ 80 years.

Bleeding events were concentrated within the first 2 days after biopsy, with 500 events on day 0 and 288 on day 1. These events accounted for 68.7% of all major bleeding events, indicating that most events occurred within the first 48 hours after biopsy. Thereafter, event frequency gradually decreased and reached a plateau (Figure 2b, Supplementary Table S2). Compared with patients aged 18–49 years, those aged ≥ 80 years showed a 4.8-fold higher observed frequency of day 0 bleeding, whereas from day 1 onward, the relative difference ranged from 5.2- to 13-fold, suggesting a possible greater susceptibility to later bleeding among older patients (Supplementary Table S2).

### Association of Clinical Factors, Age, BMI, and Prebiopsy Clinical Diagnoses With Major Bleeding Risk After PKB

The odds of major bleeding complications increased progressively with age (Figure 3a). Using the 18–49 age group as reference, the adjusted ORs were as follows: aged 50–59 years at OR, 1.71 (95% CI, 1.37–2.13); aged 60–69 years at OR, 1.85 (95% CI, 1.52–2.27); aged 70–79 years at OR, 2.89 (95% CI, 2.39–3.49); and aged ≥ 80 years at OR, 3.84 (95% CI, 3.05–4.82). Figure 3b illustrates the association between higher BMIs and reduced bleeding risk. Patients with a BMI > 25 kg/m^2^ had approximately 50% lower odds of bleeding, which may reflect a possible obesity paradox or a potential tamponade effect related to increased abdominal wall thickness.

**Figure 3:**
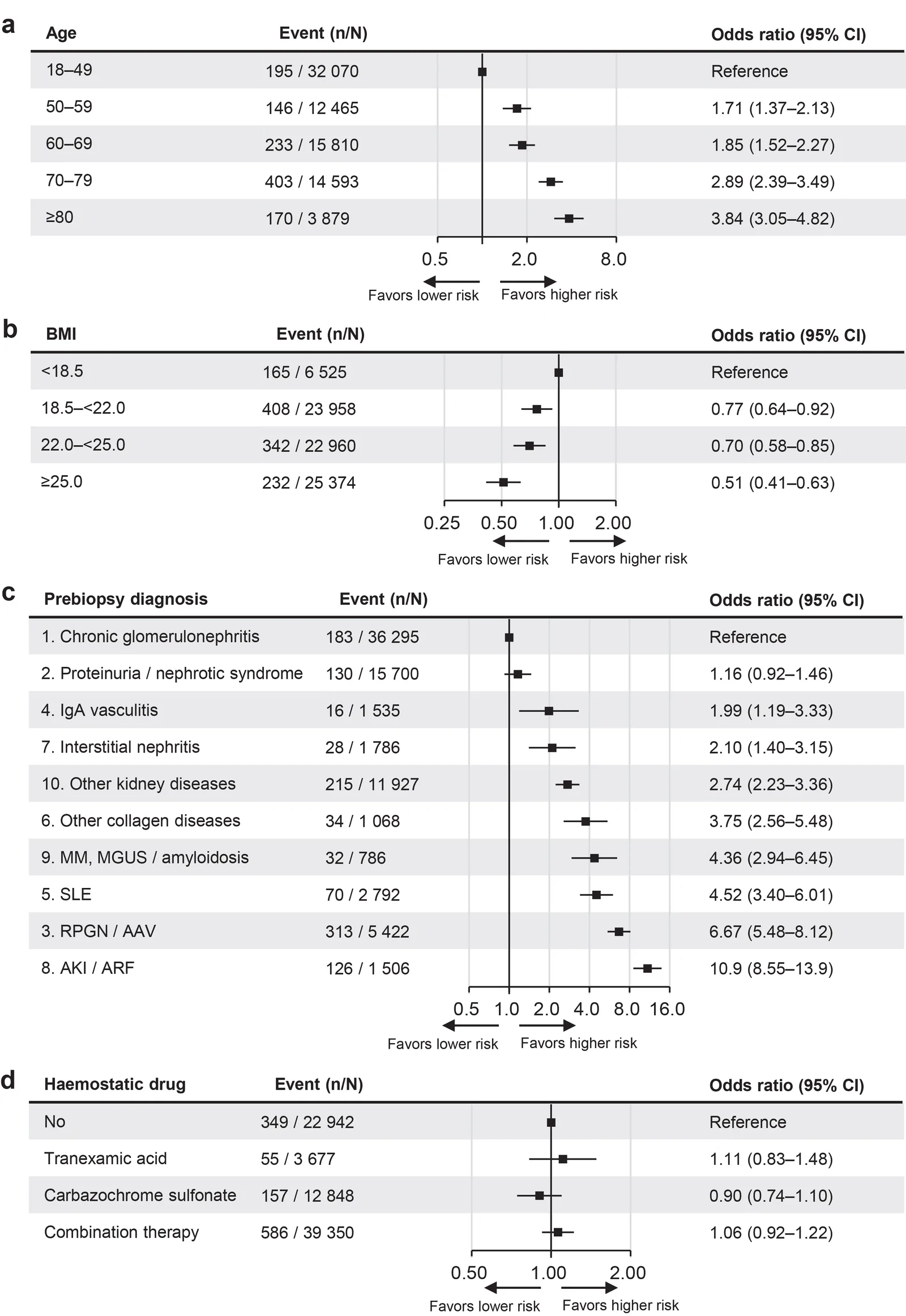
Adjusted odds ratios for major bleeding events. Forest plots showing adjusted ORs and 95% CIs for major bleeding events across different risk factors: (a) Age group (reference: 18–49 years); (b) body mass index (BMI) categories (reference: < 18.5 kg/m^2^); (c) prebiopsy clinical diagnosis categories (reference: chronic glomerulonephritis); and (d) use of hemostatic agents (reference: none). The logistic regression model was adjusted for year of biopsy, age group, sex, BMI, CCI, use of hemostatic agents, diabetes, cardiovascular disease, and prebiopsy clinical diagnostic category. AAV, antineutrophil cytoplasmic antibody–associated vasculitis; AKI, acute kidney injury; ARF, acute renal failure; BMI, body mass index; CGN, chronic glomerulonephritis; CI, confidence interval; MGUS, monoclonal gammopathy of undetermined significance; MM, multiple myeloma; OR, odds ratio; RPGN, rapidly progressive glomerulonephritis; SLE, systemic lupus erythematosus.

To evaluate the association between prebiopsy clinical diagnosis and major bleeding, patients were stratified into 10 diagnostic categories based on admission diagnosis, and the data were analyzed using multivariable logistic regression analysis (Figure 3c). Using chronic glomerulonephritis (CGN; category 1), which exhibited the lowest bleeding risk, as the reference group, the highest risks were observed in patients with rapidly progressive glomerulonephritis (RPGN) or antineutrophil cytoplasmic antibody-associated vasculitis (AAV; category 3; OR, 6.67; 95% CI, 5.48–8.12) and in those with acute kidney injury (AKI) or acute renal failure (ARF; category 8; OR, 10.9; 95% CI, 8.55–13.9). Moderately elevated risk was observed in patients with systemic lupus erythematosus (SLE; category 5; OR, 4.52; 95% CI, 3.40–6.01), and in those with multiple myeloma (MM), monoclonal gammopathy of undetermined significance (MGUS), or amyloidosis (category 9; OR, 4.36; 95% CI, 2.94–6.45). Proteinuria or nephrotic syndrome was not associated with bleeding risk (category 2; OR, 1.16; 95% CI, 0.92–1.46). The use of hemostatic agents was not significantly associated with post-PKB bleeding risk. Compared with no hemostatic agent use, tranexamic acid, carbazochrome sulfonate, or combination therapy showed no statistically significant differences in bleeding outcomes (Figure 3d). Sensitivity analyses using blood transfusion alone (Supplementary Figure S2) and transfusion of ≥ 4 units (Supplementary Figure S3) as alternative outcome definitions yielded similar results.

### Construction of the K-BIT Scoring System for Prediction of Post-PKB Bleeding Risk

#### Model Visualization and Variable Selection

After fitting the multinomial model, a 0.95 cumulative probability threshold was applied to the predicted probabilities for model visualization purposes in the test cohort (Supplementary Figures S1 and S4). At this threshold, the bleeding rate for hypothetically nonhospitalized patients (considered lower risk) was 1.24%, whereas for patients assumed to be hospitalized (considered higher risk) it was 6.35%. The postdischarge bleeding rate under this assumption was 2.85%, which was below the planned threshold of 5% (Supplementary Figure S4b).

In the derivation cohort, age category, diagnostic risk tier, BMI category, and CCI were selected for the K-BIT score based on the model-derived coefficients and clinical interpretability (Table 2 and Supplementary Table S3). The day 0 coefficients were divided by 0.3 and discretized for practical scoring. The final integer-based K-BIT score ranged from 0 to 3 points and is shown in Table 2 and Figure 4.

**Table 2:** Discretized coefficients and assigned points for selected variables in K-BIT scoring tool development

Variable	Scaled coefficient	Assigned points
Age 18–49 yr	Reference	0
Age 50–59 yr	1.08	1
Age 60–69 yr	0.67	1
Age 70–79 yr	2.64	3
Age **≥** 80 yr	3.58	3
Category 1: CGN	Reference	0
Category 2: proteinuria, NS	–0.06	0
Category 3: RPGN, AAV	5.46	3
Category 4: IgAV	1.11	1
Category 5: SLE	5.26	3
Category 6: other collagen diseases	2.45	2
Category 7: interstitial nephritis	2.03	2
Category 8: AKI, ARF	6.40	3
Category 9: MM, MGUS, amyloidosis	4.62	3
Category 10: other kidney diseases	2.34	2
BMI <18.5 kg/m^2^	Reference	2
BMI 18.5 to < 22.0 kg/m^2^	–0.43	1
BMI 22.0 to < 25.0 kg/m^2^	–0.99	0
BMI ≥ 25.0 kg/m^2^	–1.73	0
CCI score 0	Reference	0
CCI score ≥ 1		1

AAV, antineutrophil cytoplasmic antibody-associated vasculitis; AKI, acute kidney injury; ARF, acute renal failure; BMI, body mass index; CCI, Charlson Comorbidity Index; CGN, chronic glomerulonephritis; IgAV, IgA vasculitis; K-BIT, Kidney biopsy Bleeding Index for Triage; MGUS, monoclonal gammopathy of undetermined significance; MM, multiple myeloma; NS, nephrotic syndrome; RPGN, rapidly progressive glomerulonephritis; SLE, systemic lupus erythematosus.

**Figure 4:**
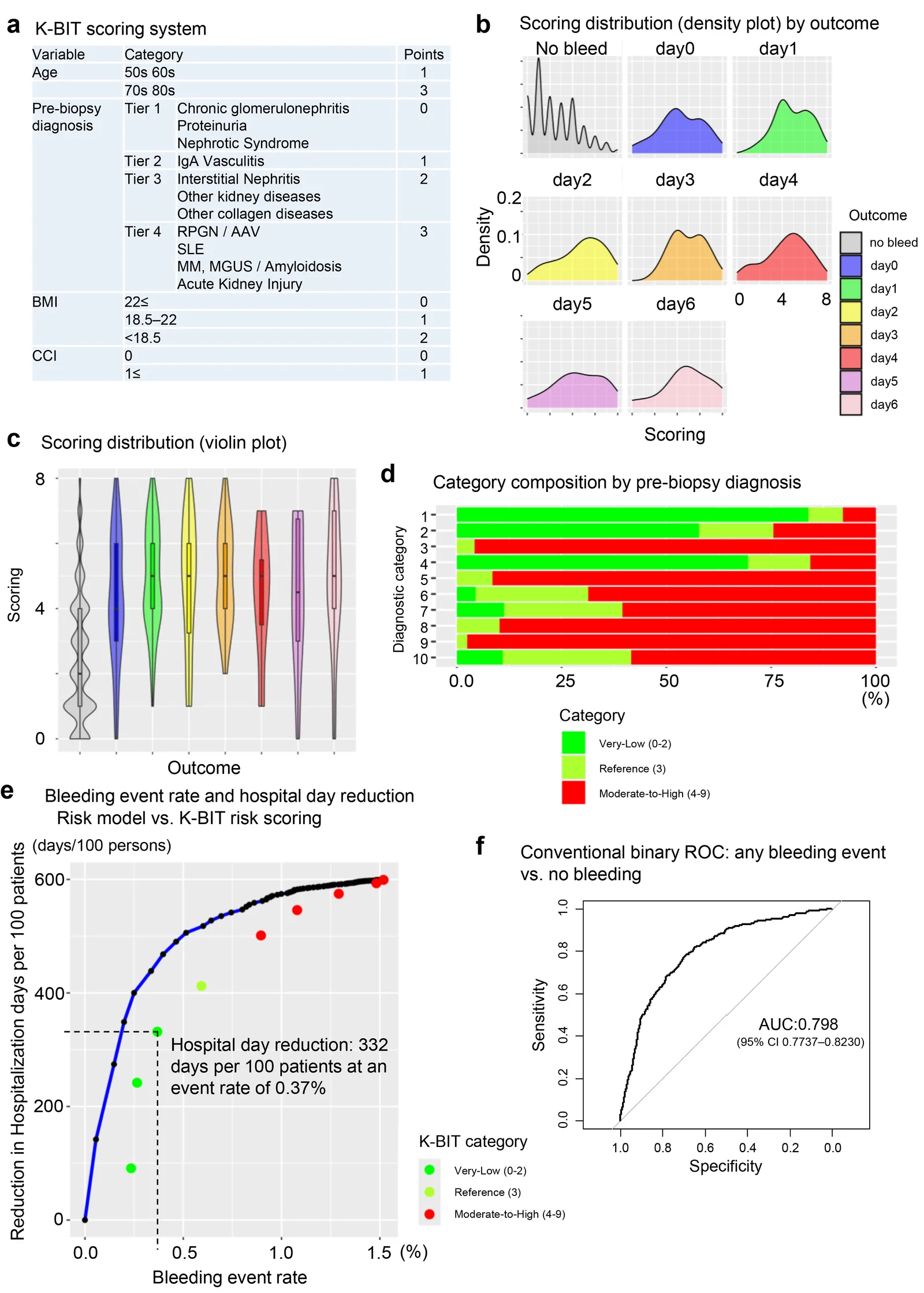
Clinical utility of the K-BIT (Kidney biopsy Bleeding Index for Triage) scoring system in the internal validation cohort. (a) Point assignment for each variable in the K-BIT Score. Table detailing the 4 prebiopsy variables (age, prebiopsy diagnosis, body mass index, and Charlson Comorbidity Index) and their corresponding integer point assignment (ranging from 0 to 3) used to calculate the final K-BIT score. (b, c) A density plot (b) and violin plot (c) showing the distribution of K-BIT scores by outcome for patients who did not or did experience a major bleeding event (stratified by the day of event: day 0 to day 6). The figure illustrates the score’s discriminatory ability, with bleeding events clustering at higher scores. (d) Category composition by group. Bar chart illustrating the 3 K-BIT risk tiers within each prebiopsy diagnostic category: very-low-risk (0–2), reference-risk (3), and moderate-to-high-risk (4–9). (e) Plot of the bleeding event rate and hospital day reduction comparing predicted bleeding event rates (%) against the reduction in hospitalization days per 100 patients, achieved by applying K-BIT-based discharge strategies. The smooth blue curve represents predicted-risk thresholds evaluated in the simulation model to illustrate the trade-off between estimated hospitalization days saved and bleeding event rates. As a reference, discharging all patients on the same day as PKB would result in a bleeding event rate equal to the cohort average of 1.52%, represented by the red dot in the upper-right corner of the figure. The figure suggests that restricting early discharge to patients with scores ≤ 2 (very-low-risk in K-BIT) achieved a low event rate (0.37%) and an estimated reduction in hospital stay (332 days per 100 patients). (f) Conventional ROC with dichotomized outcome as bleeding event versus no bleeding. The vertical axis represents sensitivity, and the horizontal axis represents specificity. The model demonstrated good discrimination with an AUC of 0.7984 (95% CI, 0.7737–0.8230). AUC, area under the curve; CI, confidence interval; ROC, receiver operating characteristics.

#### Score Validation and Risk Stratification

The observed K-BIT scores for the validation cohort ranged from 0 to 8 (Supplementary Table S4). The scoring system demonstrated strong discriminatory ability; the event rate was 0.23% in the lowest score group (score 0) and gradually increased with higher scores (Supplementary Table S4). Nonbleeding patients showed a left-skewed distribution toward lower scores, whereas bleeding events clustered at higher scores (Figure 4b and c).

We defined 3 risk tiers: very-low-risk (scores 0–2), reference-risk (score 3), and moderate-to-high-risk (scores ≥ 4) groups (Figure 4d). Very-low-risk patients (scores 0–2) comprised 55.4% of the validation cohort and had a major bleeding rate of 0.37%, which is substantially lower than the cohort average of 1.52%. The reference-risk group (score 3) was defined as the score category in which the observed bleeding incidence was comparable to that of the overall cohort. Higher score categories were then interpreted as representing progressively elevated bleeding risk. Patients with scores ≥ 4 accounted for 31.2% of the full cohort, and their observed bleeding incidence rate was 3.56%, confirming the score’s utility for identifying high-risk patients (Supplementary Table S4).

### Clinical Impact and Hospitalization Reduction

Simulation analysis suggested substantial resource-saving potential under hypothetical discharge strategies. We assessed early discharge thresholds across predicted bleeding risk levels to optimize the trade-off between hospitalization days saved and bleeding event rates (Figure 4e). In the simulation, restricting early discharge to patients with scores ≤ 2 would be associated with an estimated event rate of 0.37% and a reduction of 332 hospital days per 100 patients, compared with approximately 600 hospital days under the conventional 6-day hospitalization practice in Japan (55% reduction; Figure 4e). These findings indicate substantial resource savings while maintaining a low risk of postdischarge bleeding. Finally, when bleeding outcomes were dichotomized as any bleeding event versus no bleeding, the model demonstrated good discrimination, with an AUC of 0.7984 (95% CI, 0.7737–0.8230; Figure 4f).

## Discussion

We developed and validated the K-BIT, a simple scoring system for estimating early bleeding risk based solely on 4 clinical variables readily available before admission, namely age, diagnostic category, BMI, and CCI. The score successfully stratified bleeding risk in a non-overlapping test cohort, and simulation analysis suggested the potential to reduce hospital length of stay while maintaining patient safety. In fact, more than half of patients (55.4%) had K-BIT scores ≤ 2 and their bleeding event rate was as low as 0.37%. Restricting the scoring variables to clinical information that can be collected from patient interviews alone enhances its applicability. The timing of bleeding events also supports its potential relevance for risk-based discharge strategies, as 68.7% of major bleeding events occurred within the first 2 days after biopsy.

In this nationwide cohort of 78,817 adults undergoing PKB in Japan, the overall incidence of major bleeding complications was 1.46%. Risk increased with age and decreased with increasing BMI, and varied by diagnosis, being highest in RPGN/AAV and AKI/ARF and lowest in CGN. Among the variables commonly evaluated in prediction scores or models, age and BMI have been among the most frequently assessed and incorporated measures in previous studies.^17^, ^34^, ^38^, ^39^, ^40^ Consistent with previous findings,^38^, ^39^, ^40^ older age was associated with increased risk of major bleeding events after PKB in our nationwide cohort. Regarding BMI, previous studies have generally suggested that higher BMI is associated with a lower risk of bleeding,^17^, ^19^, ^41^ or at least is not associated with an increased risk of postbiopsy complications.^34^, ^42^ In our study, lower BMI showed a graded association with a higher risk of bleeding throughout the first week after PKB (Supplementary Table S3). Diagnosis-specific risk stratification further supports individualized decision-making. Bleeding risk was high in patients with RPGN/AAV, AKI, and ARF. The increased risk in AKI/ARF has been previously documented,^6^, ^34^ whereas evidence for RPGN/AAV is limited. This elevated risk may be attributed to the underlying severe renal impairment and systemic inflammation accompanying these conditions. Despite the suboptimal systemic conditions, both RPGN/AAV and AKI/ARF represent clinical emergencies that often necessitate urgent kidney biopsy. Furthermore, RPGN/AAV are often associated with anemia due to systemic inflammation. Conversely, patients with CGN and those presenting with proteinuria or nephrotic syndrome exhibited relatively low bleeding risk, supporting the rationale for active PKB in these groups.^43^ Systemic vasculitis, such as IgA vasculitis, showed a higher bleeding risk than CGN. These findings highlight the importance of incorporating clinical diagnosis into risk assessment models before PKB, facilitating better-informed decision-making and tailored patient counseling.

Several tools have been reported to estimate bleeding risk after PKB (Supplementary Table S5). Among them, the calculator developed by Schorr *et al.* has shown good discriminatory performance (C-statistic 0.83), although its development cohort was relatively small.^16^, ^17^, ^18^, ^19^, ^20^ Halimi *et al.*^21^ also introduced a nationwide French claims-based risk score that was later simplified (receiver operating characteristic AUC 0.755 [95% CI, 0.746–0.763]). The Halimi score incorporated diagnosis-related categories, laboratory data, and clinical variables and represented an important advance in risk stratification using nationwide administrative data. However, the primary objective of these models was prediction of bleeding risk itself.^22^ In contrast, K-BIT was specifically developed as a preadmission triage tool to support decisions regarding observation intensity and same-day discharge and incorporates prebiopsy clinical diagnostic categories routinely available in nephrology practice. To maximize practicality, K-BIT relies exclusively on information available before admission and does not require laboratory or imaging data. In addition, our modeling framework incorporated the timing of bleeding events, allowing assessment of early postbiopsy risk most relevant to discharge planning. Finally, we evaluated the potential impact of score-guided management on hospitalization burden, extending the model beyond risk prediction alone.

Although prebiopsy diagnosis may introduce some inherent bias, it is part of the clinical context rather than an additional requirement, making the score practical and broadly applicable. We assigned the highest score to prebiopsy diagnostic tier 4 (RPGN/AAV, SLE, MM, MGUS, or amyloidosis, and AKI; Table 2 and Figure 4a). Given the expected low frequency of misclassification among tier 4 diagnoses, diagnostic misclassification is unlikely to substantially compromise either the safety or performance of the scoring system. As for the overall incidence, the rate of major bleeding events was 1.46%; 1091 (1.38%) patients required transfusion, 109 (0.14%) underwent surgical or endovascular interventions, and no patients required nephrectomy, which is consistent with previously reported ranges despite variability in outcome definitions across studies.^17^, ^34^, ^44^ The relatively low incidence observed in our cohort is likely attributable to our stringent definition of major bleeding, which was limited to transfusion or surgical or interventional hemostasis and did not include minor bleeding complications managed conservatively. Finally, we incorporated what is likely the most detailed and wide-ranging diagnostic classification used in this field.^21^

This study has several strengths. A major strength of this study is the inclusion of a substantial number of patients undergoing PKB, which enabled us to evaluate the association between prebiopsy clinical diagnosis and postbiopsy bleeding risk despite the low incidence of the outcome. The large nationwide cohort ensures that this study reflects real-world clinical practice across diverse hospitals and geographic regions throughout Japan. Another strength is that the unique routine hospitalization practice after PKB in Japan allowed us to assess bleeding events over the entire first week after the procedure, thereby capturing both immediate complications and delayed bleeding events that may be missed in studies with shorter observation periods. This setting provided a unique opportunity to comprehensively characterize postbiopsy bleeding patterns and develop a risk-stratification framework that may be informative across diverse health care systems and observation strategies. Moreover, bleeding events were evaluated according to their timing rather than being treated solely as a binary outcome, providing additional insight into the temporal pattern of PKB bleeding. Finally, we assessed the performance of the risk model using not only conventional discrimination measures such as the AUC, but also measures that accounted for the balance between clinical benefit and potential harm, which is particularly important and more appropriate than discrimination measures alone given the rarity of major bleeding events after PKB. Through this approach, albeit in a simulation-based analysis, this study extends beyond risk prediction alone by linking the findings to a potential change in clinical practice and more efficient use of health care resources.

However, the study also has several limitations. First, because laboratory and imaging data were unavailable, we could not evaluate the incremental predictive value of established bleeding-risk predictors such as hemoglobin level, platelet count, coagulation parameters, kidney function, or kidney size. Administrative diagnosis codes may partially capture conditions such as anemia or chronic kidney disease; however, these variables do not adequately reflect disease severity and cannot serve as direct substitutes for laboratory measurements. Therefore, the model should be considered a simplified prebiopsy risk stratification tool, although incorporation of laboratory variables may further improve predictive performance. Second, detailed information on biopsy techniques (e.g., needle gauge, number of passes, and histologic findings) was unavailable. Third, because the administration of hemostatic agents was not standardized but rather reflected real-world practice patterns in Japan, confounding by indication cannot be entirely excluded.^8^ Fourth, diagnosis and procedure codes in the Diagnosis Procedure Combination database may be prone to misclassification despite using validated definitions. Fifth, the estimated reduction in hospital length of stay was based on a hypothetical management simulation rather than direct evaluation in clinical practice. Therefore, these estimates should be interpreted as model-based projections rather than demonstrated clinical effects.

For transparency, we note that a preliminary version of this work was previously reported as a conference abstract.^45^ After abstract submission, tumor-related biopsy cases were excluded after refinement of the eligibility criteria, and an error in kidney-biopsy date assignment within the merged database was subsequently identified and corrected. All analyses were repeated using the corrected dataset. The corrected dataset formed the basis of both the WCN26 conference presentation and the current manuscript.^45^

Finally, external validation using an independent cohort was not performed, and further validation studies are needed to confirm the generalizability of the findings. To fully understand the true utility of this simple scoring system, future studies should examine how the K-BIT score, with or without pre- and postprocedural laboratory data and ultrasound monitoring, is associated with improved patient outcomes, using information from real clinical records. To support clinical implementation of the K-BIT score, a freely accessible web-based calculator (version 1.0.1) is available at https://rena-med.github.io/kbit-score/.

## Conclusion

This large-scale nationwide study in Japan demonstrated that major bleeding complications after PKB vary substantially by age and prebiopsy clinical diagnosis. By integrating readily available prebiopsy variables into a simple triage tool, K-BIT enables risk-based stratification and may support individualized decisions regarding the postbiopsy monitoring intensity, follow-up strategy, and observation duration. In real-world clinical practice, this model could be used in combination with laboratory findings to improve the identification of patients at risk for major bleeding after PKB, thereby supporting changes in clinical practice that promote the appropriate use of health care resources.

## Disclosures

All the authors declared no competing interests.

## Supplementary Material

Supplementary File (PDF) **Figure S1.** Flowchart of model development and score generation. **Figure S2.** Adjusted odds ratios for blood transfusion across clinical and treatment-related factors. **Figure S3.** Adjusted odds ratios for ≥4-unit transfusion across clinical and treatment-related factors. **Figure S4.** Descriptive visualization of model performance at a 0.95 threshold and bleeding rates across predicted outcome groups. **Table S1.** Mapping of ICD-10 codes to pre-biopsy diagnostic categories. **Table S2.** Bleeding events by age group. **Table S3.** Coefficients from the L2-penalized multinomial logistic regression model by bleeding-timing category. **Table S4.** Distribution of K-BIT scores and associated bleeding events in the internal validation cohort. **Table S5.** Summary of variables used in existing bleeding risk scores. STROBE Checklist. TRIPOD Checklist.

## Footnotes

Supplementary File (PDF)
**Figure S1.** Flowchart of model development and score generation. **Figure S2.** Adjusted odds ratios for blood transfusion across clinical and treatment-related factors. **Figure S3.** Adjusted odds ratios for ≥4-unit transfusion across clinical and treatment-related factors. **Figure S4.** Descriptive visualization of model performance at a 0.95 threshold and bleeding rates across predicted outcome groups. **Table S1.** Mapping of ICD-10 codes to pre-biopsy diagnostic categories. **Table S2.** Bleeding events by age group. **Table S3.** Coefficients from the L2-penalized multinomial logistic regression model by bleeding-timing category. **Table S4.** Distribution of K-BIT scores and associated bleeding events in the internal validation cohort. **Table S5.** Summary of variables used in existing bleeding risk scores. STROBE Checklist. TRIPOD Checklist.
