# Supplementary material for: Creation of a Pre-biopsy Clinical Risk Score for Major Bleeding After Kidney Biopsy
Source: Kidney Int Rep. 2026 Oct 1;11(12):107118. doi: 10.1016/j.ekir.2026.107118 (PMC13733331; doi:10.1016/j.ekir.2026.107118)
Supplement: Supplementary file 1 [file mmc1.pdf]

Development and Held-out Validation of a Pre-biopsy Clinical Risk Score for Major Bleeding After Kidney Biopsy: A Nationwide Study of 78,817 Cases

Rena Suzukawa[1†], Daiei Takahashi[1,2], Takanori Hasegawa[3*], Shunsuke Inaba[1], Ken Ikenouchi[1], Yuta Nakano[1,4], Yurina Yataka[2], Mizuki Watada[2], Motoki Hoshino[2], Wakana Shoda[2], Tamaki Kuyama[2], Eisei Sohara[1], Kiyohide Fushimi[3], and Shinichi Uchida[1], and Shintaro Mandai[1*]

[1]Department of Nephrology, Graduate School of Medical and Dental Sciences, Institute of Science Tokyo, 1-5-45 Yushima, Bunkyo, Tokyo 113-8510, Japan
[2]Department of Nephrology, Musashino Red Cross Hospital, 1-26-1, Kyonann-cho, Musashino-shi, Tokyo, 180-8610, Japan
[3] M&D Data Science Centre, Institute of Integrated Research, Institute of Science Tokyo, 1-5-45 Yushima, Bunkyo, Tokyo 113-8510, Japan
[4]Population Health Sciences, University of Bristol, Bristol, Canynge Hall, 39 Whatley Road, Bristol, BS8 2PS, UK

[†] Suzukawa R is the first author of this study.

***Corresponding Authors**:
Shintaro Mandai and Takanori Hasegawa
Department of Nephrology, Graduate School of Medical and Dental Sciences, Tokyo Medical and Dental University, 1-5-45 Yushima, Bunkyo, Tokyo 113-8510, Japan
Tel: +81-3-5803-5214; Fax: +81-3-5803-5215; E-mail: smandai.kid@tmd.ac.jp and tk.hasegawa@gmail.com

Supplementary Methods

Modelling design and visualization

A multinomial model was chosen rather than an ordinal logistic regression model because postoperative bleeding timing was not assumed to represent a monotonic ordinal severity scale, and the non-bleeding category was considered qualitatively distinct from bleeding-day categories. The regularization parameter was selected through 10-fold cross-validation. Thereafter, for descriptive visualization of the model in test cohort, each subject was assigned to the first category for which the cumulative predicted probability >95%. The threshold was solely for visualization and did not affect coefficient estimation. Patients were assumed to remain hospitalized until the day after the predicted bleeding day. The resulting model-assigned hospitalization management categories were compared with actual bleeding outcomes.

Sensitivity Analyses

To assess the robustness of the primary findings, two sensitivity analyses were performed. First, when major bleeding was limited to the occurrence of blood transfusion, the results were largely unchanged from those shown in Figure 3, as the vast majority of outcome observations in the primary analysis satisfied this restriction (Figure S2). Second, a stricter threshold of ≥4 units of initial transfusion dose was used as an alternative outcome. The direction and magnitude of associations for age, BMI, and all diagnosis categories remained consistent (Figure S3). Given the limited sample size in the restricted cohort, the results should be interpreted as exploratory.

Supplementary Table 1. Mapping of ICD-10 codes to pre-biopsy diagnostic categories

ICD-10 code	Disease	Category
**T86.1**	Kidney transplant failure and rejection	Exclusion criteria
**Z94.0**	Kidney transplant status	
**N02**	Recurrent and persistent haematuria	Category1. Chronic glomerulonephritis
**N03**	Chronic nephritic syndrome	
**N05**	Unspecified nephritic syndrome	
**N04**	Nephrotic syndrome	Category2: Proteinuria and nephrotic syndrome
**N39.1**	Persistent proteinuria, unspecified	
**R80**	Isolated proteinuria	
**N06**	Isolated proteinuria with specified morphological lesion	
**M31.0**	Hypersensitivity angiitis (including Goodpasture syndrome)	Category 3: Rapidly progressive glomerulonephritis, ANCA-associated vasculitis
**M31.1**	Thrombotic microangiopathy	
**M31.3**	Wegener granulomatosis	
**M31.4**	Aortic arch syndrome [Takayasu]	
**M31.5**	Giant cell arteritis with polymyalgia rheumatica	
**M31.6**	Other giant cell arteritis	
**M31.7**	Microscopic polyangiitis	
**M31.8**	Other specified necrotizing vasculopathies	
**M31.9**	Necrotizing vasculopathy, unspecified	
**N01**	Rapidly progressive nephritic syndrome (including anti-glomerular basement membrane disease)	
**M30.1**	Polyarteritis with lung involvement [Churg-Strauss]	
**D69.0**	Allergic purpura	Category 4: IgA vasculitis
**N08.2**	Glomerular disorders in blood diseases and disorders involving the immune mechanism [IgA vasculitis]	
**M32**	Systemic lupus erythematosus (SLE)	Category 5: SLE
**N08.5**	Glomerular disorders in systemic connective tissue disorders (including lupus nephritis)	
**M30.0**	Polyarteritis nodosa	Category 6: Other collagen diseases
**M30.2**	Juvenile polyarteritis	

**M30.3**	Mucocutaneous lymph node syndrome [Kawasaki]	
**M30.8**	Other conditions related to polyarteritis nodosa	
**M33.0**	Juvenile dermatomyositis	
**M33.1**	Other dermatomyositis	
**M33.2**	Polymyositis	
**M33.9**	Dermatopolymyositis, unspecified	
**M34.0**	Progressive systemic sclerosis	
**M34.1**	CR(E)ST syndrome	
**M34.2**	Systemic sclerosis induced by drugs and chemicals	
**M34.8**	Other forms of systemic sclerosis	
**M34.9**	Systemic sclerosis, unspecified	
**M35.0**	Sicca syndrome [Sjögren]	
**M35.1**	Other overlap syndromes	
**M35.2**	Behçet disease	
**M35.3**	Polymyalgia rheumatica	
**M35.4**	Diffuse (eosinophilic) fasciitis	
**M35.5**	Multifocal fibrosclerosis	
**M35.6**	Relapsing panniculitis [Weber-Christian]	
**M35.7**	Hypermobility syndrome	
**M35.8**	Other specified systemic involvement of connective tissue	
**M35.9**	Systemic involvement of connective tissue, unspecified	
**M36.0**	Dermato(poly)myositis in neoplastic disease	
**M36.1**	Arthropathy in neoplastic disease	
**M36.2**	Haemophilic arthropathy	
**M36.3**	Arthropathy in other blood disorders	
**M36.4**	Arthropathy in hypersensitivity reactions classified elsewhere	
**M36.8**	Systemic disorders of connective tissue in other diseases classified elsewhere	
**N10**	Acute tubulo-interstitial nephritis	Category 7: Interstitial nephritis
**N11**	Chronic tubulo-interstitial nephritis	

**N12**	Tubulo-interstitial nephritis, not specified as acute or chronic	
**N17**	Acute renal failure	Category 8: Acute kidney injury, acute renal failure
**N28.8**	Other specified disorders of kidney and ureter [Acute kidney Injury]	
**C90.0**	Multiple myeloma	Category 9: Multiple myeloma, MGUS, amyloidosis
**E85**	Amyloidosis	
**N29.8**	Other disorders of kidney and ureter in other diseases classified elsewhere (including renal amyloidosis)	
**D47.2**	Monoclonal gammopathy of undetermined significance (MGUS)	
**E10.2**	Type 1 diabetes mellitus with renal complications	Category 10: Other kidney diseases
**E10.7**	Type 1 diabetes mellitus with multiple complications	
**E11.2**	Type 2 diabetes mellitus with renal complications	
**E11.7**	Type 2 diabetes mellitus with multiple complications	
**E12.2**	Malnutrition-related diabetes mellitus with renal complications	
**E12.7**	Malnutrition-related diabetes mellitus with multiple complications	
**E13.2**	Other specified diabetes mellitus with renal complications	
**E13.7**	Other specified diabetes mellitus with multiple complications	
**E14.2**	Unspecified diabetes mellitus with renal complications	
**E14.7**	Unspecified diabetes mellitus with multiple complications	
**I12.0**	Hypertensive renal disease with renal failure	
**I12.9**	Hypertensive renal disease without renal failure	
**N00**	Acute nephritic syndrome	
**N07**	Hereditary nephropathy, not elsewhere classified	

**N08.0**	Glomerular disorders in infectious and parasitic diseases classified elsewhere	
**N08.1**	Glomerular disorders in neoplastic diseases	
**N08.3**	Glomerular disorders in diabetes mellitus	
**N08.4**	Glomerular disorders in other endocrine, nutritional and metabolic diseases	
**N08.8**	Glomerular disorders in other diseases classified elsewhere	
**N14**	Drug- and heavy-metal-induced tubulo-interstitial and tubular conditions	
**N15.8**	Other specified renal tubulo-interstitial diseases	
**N15.9**	Renal tubulo-interstitial disease, unspecified	
**N16**	Renal tubulo-interstitial disorders in diseases classified elsewhere	
**N18**	Chronic kidney disease	
**N19**	Unspecified kidney failure	
**N25**	Disorders resulting from impaired renal tubular function	
**N26**	Unspecified contracted kidney	
**N28.9**	Disorder of kidney and ureter, unspecified	
**R31**	Unspecified haematuria	

Supplementary Table 2. Bleeding events by age group

	Day0	Day1	Day2	Day3	Day4	Day5	Day6	No bleed	Total
**≥80**	63 (1.62%)	47 (1.21%)	26 (0.67%)	11 (0.28%)	6 (0.15%)	12 (0.31%)	5 (0.13%)	3 709 (95.62%)	3 879
**70-79**	169 (1.16%)	113 (0.77%)	41 (0.28%)	33 (0.23%)	13 (0.09%)	16 (0.11%)	18 (0.12%)	14 190 (97.24%)	14 593
**60-69**	82 (0.52%)	63 (0.4%)	30 (0.19%)	21 (0.13%)	13 (0.08%)	15 (0.09%)	9 (0.06%)	15 577 (98.53%)	15 810
**50-59**	77 (0.62%)	35 (0.28%)	15 (0.12%)	5 (0.04%)	4 (0.03%)	4 (0.03%)	6 (0.05%)	12 319 (98.83%)	12 465
**18-49**	109 (0.34%)	30 (0.09%)	19 (0.06%)	13 (0.04%)	7 (0.02%)	9 (0.03%)	8 (0.02%)	31 875 (99.39%)	32 070
**Total**	500	288	131	83	43	56	46	77 670	**78 817**

Tabular summary of Figure 2b. Data are presented as n (%). Percentages are calculated within each age category.

Supplementary Table 3. Coefficients from the L2-penalized multinomial logistic regression model by bleeding-timing category

Variable		Day 0	Day 1	Day 2	Day 3	Day 4	Day 5	Day 6
Age	18–49	Reference						
	50–59	0.324	0.238	0.224	0.116	0.11	0.14	0.123
	60–69	0.2	0.376	0.408	0.421	0.353	0.375	0.254
	70–79	0.791	1.001	0.636	0.688	0.629	0.569	0.762
	80–	1.075	1.139	1.159	1.015	1.04	1.265	0.842
Female		0.144	0.346	0.271	0.161	0.123	0.083	0.131
Year	2016–2017	Reference						
	2018–2019	0.035	0.101	0.18	0.034	0.02	0.072	-0.036
	2020–2021	0.223	0.173	0.098	0.036	0.002	0.066	0.064
CCI score		0.196	0.272	0.219	0.199	0.231	0.245	0.237
Haemostatic drug		0.083	-0.051	-0.02	0.014	-0.062	-0.016	0.008
Diagnostic category	1	Reference						
	2	-0.017	-0.214	-0.024	-0.143	-0.144	0.047	-0.177
	3	1.637	1.688	1.631	1.715	1.532	1.508	1.586
	4	0.334	0.107	0.16	0.308	0.101	0.085	0.628
	5	1.579	0.605	0.585	0.696	0.634	0.612	0.638
	6	0.734	1.384	0.139	0.951	0.749	0.716	0.418
	7	0.61	0.219	0.442	0.297	0.148	0.127	0.575
	8	1.92	2.154	2.069	2.511	2.667	1.815	2.207
	9	1.386	1.218	0.644	1.855	1.08	0.615	1.093
	10	0.702	0.688	0.766	0.537	0.714	0.678	0.591
BMI, kg/m^2	<18.5	Reference						
	18.5-<22.0	-0.128	-0.147	-0.066	-0.173	-0.051	-0.075	-0.108
	22.0-<25.0	-0.297	-0.288	-0.161	-0.176	-0.188	-0.135	-0.124
	≥25.0	-0.518	-0.444	-0.486	-0.281	-0.446	-0.391	-0.408

Age, sex, year of biopsy, use of haemostatic agents, prebiopsy clinical diagnosis category, BMI, and hospital size were entered into the model as categorical variables, and CCI score was entered as a continuous variable. BMI, body mass index; CCI, Charlson Comorbidity Index.

Supplementary Table 4. Distribution of K-BIT scores and associated bleeding events in the internal validation cohort

	Score								
Reference	0	1	2	3	4	5	6	7	8
No bleeding	2977	4920	2927	2599	2846	1417	891	563	184
Bleeding on day 0	5	8	11	23	32	17	24	16	5
Bleeding on day 1	0	1	3	6	17	9	12	10	2
Bleeding on day 2	0	3	2	3	3	7	5	6	1
Bleeding on day 3	0	0	1	4	8	4	7	4	1
Bleeding on day 4	0	1	0	1	1	2	1	1	0
Bleeding on day 5	1	1	1	3	3	3	1	5	0
Bleeding on day 6	1	0	1	0	3	4	0	3	1
Bleeding rate, %	0.23	0.28	0.64	1.52	2.30	3.14	5.31	7.40	5.15
Cumulative bleeding event, %	0.23	0.27	0.37	0.59	0.90	1.08	1.29	1.48	**1.52**

Distribution of K-BIT scores and observed bleeding events in the validation cohort. Each cell shows the number of patients with a given score who experienced bleeding on the corresponding post-biopsy day. The bleeding event rate for each score category and the cumulative bleeding event rate are also shown on the bottom. The overall incidence of bleeding events was 1.52%.

Supplementary Table 5. Summary of variables used in existing bleeding risk scores

Variable	KBRC Schorr et al.	Mejía-Vilet et al.	Halimi et al.	K-BIT score
Age	Yes			Yes
Sex			Yes	
Body mass index	Yes		Yes	Yes
Dyslipidaemia			Yes	
Comorbidities / Charlson Comorbidity Index			Yes	Yes
Frailty			Yes	
Cancer			Yes	
Haemoglobin	Yes	Yes	Yes	
Platelet count	Yes	Yes	Yes	
Serum creatinine / eGFR			Yes	
Blood urea nitrogen		Yes		
Pre- or post-ultrasonography	Yes	Yes		
Antithrombotic medication history	No	No	No	No
Haemostatic medication use	No	No	No	No
Requires laboratory data	Yes	Yes	Yes	No
Requires imaging data	Yes	Yes		No
Complex diagnostic coding required			Yes	
Preadmission risk stratification possible	Partial	No	Partial	Yes

Comparison of variables included in published risk scores for post-percutaneous kidney biopsy bleeding. The table indicates whether each scoring requires specific clinical, laboratory, imaging, or operational variables (Yes/No).

Supplementary Figures

Figure S1

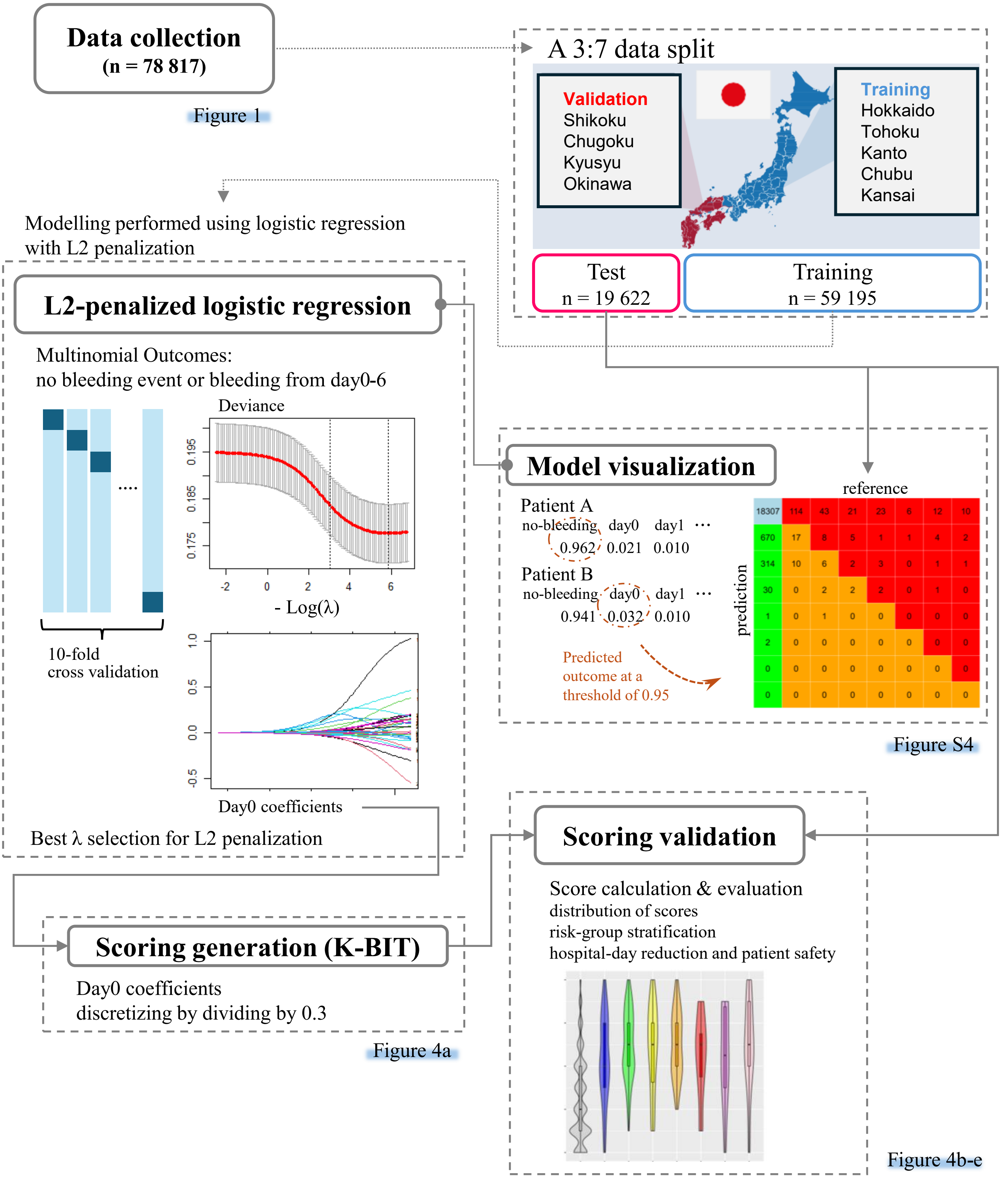
Data collection
(n = 78 817)
Figure 1
A 3:7 data split
Validation
Shikoku
Chugoku
Kyusyu
Okinawa
Training
Hokkaido
Tohoku
Kanto
Chubu
Kansai
Test
n = 19 622
Training
n = 59 195
Modelling performed using logistic regression with L2 penalization
L2-penalized logistic regression
Multinomial Outcomes:
no bleeding event or bleeding from day0-6
Deviance
- Log(λ)
10-fold cross validation
Day0 coefficients
Best λ selection for L2 penalization
Model visualization
Patient A
no-bleeding day0 day1 ···
0.962 0.021 0.010
Patient B
no-bleeding day0 day1 ···
0.941 0.032 0.010
Predicted outcome at a threshold of 0.95
reference
prediction
Figure S4
Scoring generation (K-BIT)
Day0 coefficients
discretizing by dividing by 0.3
Figure 4a
Scoring validation
Score calculation & evaluation
distribution of scores
risk-group stratification
hospital-day reduction and patient safety
Figure 4b-e

Figure S1. Flowchart of model development and score generation

Patients were divided into training and test (validation) datasets. The training dataset was used for model construction using L2-penalized logistic regression, and descriptive visualization of the model using the test data was performed using a cumulative probability threshold of 0.95. Day 0 coefficients were discretized by dividing them by 0.3 to generate the scoring system. The score distribution, risk-group stratification, reduction in hospitalization days, and safety outcomes were subsequently assessed using a non-overlapping held-out test cohort derived from the same dataset.

Figure S2

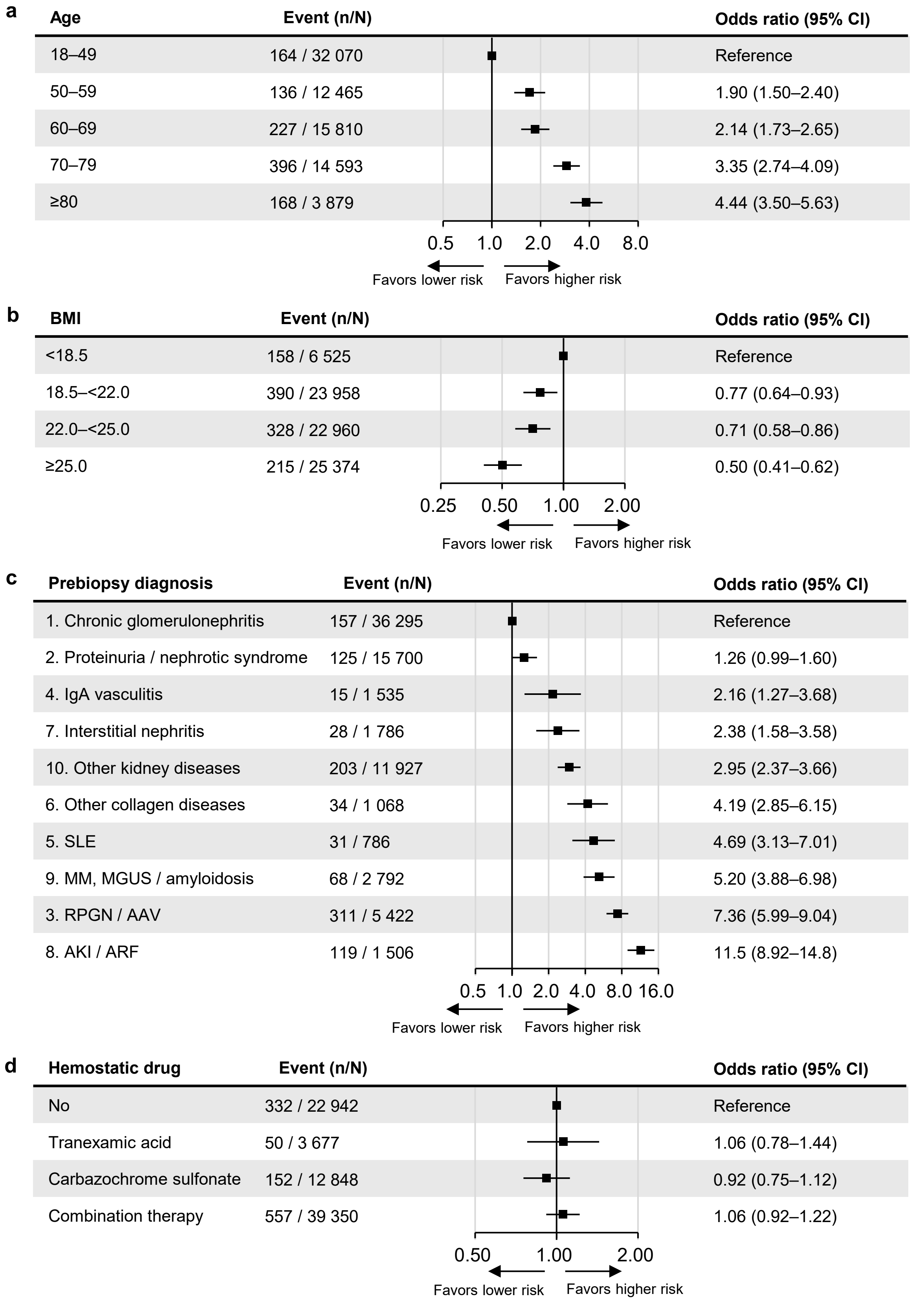
a
Age
Event (n/N)
Odds ratio (95% CI)
18–49
164 / 32 070
Reference
50–59
136 / 12 465
1.90 (1.50–2.40)
60–69
227 / 15 810
2.14 (1.73–2.65)
70–79
396 / 14 593
3.35 (2.74–4.09)
≥80
168 / 3 879
4.44 (3.50–5.63)
0.5
1.0
2.0
4.0
8.0
Favors lower risk
Favors higher risk
b
BMI
Event (n/N)
Odds ratio (95% CI)
<18.5
158 / 6 525
Reference
18.5–<22.0
390 / 23 958
0.77 (0.64–0.93)
22.0–<25.0
328 / 22 960
0.71 (0.58–0.86)
≥25.0
215 / 25 374
0.50 (0.41–0.62)
0.25
0.50
1.00
2.00
Favors lower risk
Favors higher risk
c
Prebiopsy diagnosis
Event (n/N)
Odds ratio (95% CI)
1. Chronic glomerulonephritis
157 / 36 295
Reference
2. Proteinuria / nephrotic syndrome
125 / 15 700
1.26 (0.99–1.60)
4. IgA vasculitis
15 / 1 535
2.16 (1.27–3.68)
7. Interstitial nephritis
28 / 1 786
2.38 (1.58–3.58)
10. Other kidney diseases
203 / 11 927
2.95 (2.37–3.66)
6. Other collagen diseases
34 / 1 068
4.19 (2.85–6.15)
5. SLE
31 / 786
4.69 (3.13–7.01)
9. MM, MGUS / amyloidosis
68 / 2 792
5.20 (3.88–6.98)
3. RPGN / AAV
311 / 5 422
7.36 (5.99–9.04)
8. AKI / ARF
119 / 1 506
11.5 (8.92–14.8)
0.5
1.0
2.0
4.0
8.0
16.0
Favors lower risk
Favors higher risk
d
Hemostatic drug
Event (n/N)
Odds ratio (95% CI)
No
332 / 22 942
Reference
Tranexamic acid
50 / 3 677
1.06 (0.78–1.44)
Carbazochrome sulfonate
152 / 12 848
0.92 (0.75–1.12)
Combination therapy
557 / 39 350
1.06 (0.92–1.22)
0.50
1.00
2.00
Favors lower risk
Favors higher risk

Figure S2. Adjusted odds ratios for blood transfusion across clinical and treatment-related factors.

This figure presents forest plots showing adjusted odds ratios (ORs) and 95% confidence intervals (CIs) for the risk of red blood cell transfusion following percutaneous kidney biopsy. Estimates were derived from multivariable logistic regression models adjusted for year of biopsy, age group, sex, body mass index (BMI), Charlson Comorbidity Index, diabetes, cardiovascular disease, and prebiopsy clinical diagnosis. **(a)** age-stratified estimates using the 18–49-year group as the reference. **(b)** BMI-stratified estimates with <18.5 kg/m² as the reference. **(c)** shows results across prebiopsy clinical diagnosis categories relative to chronic glomerulonephritis. **(d)** represents the association between haemostatic agent use and transfusion risk, with no haemostatic therapy as the reference category.

OR, odds ratio; CI, confidence interval; CGN, chronic glomerulonephritis; SLE, systemic lupus erythematosus; MM, multiple myeloma; MGUS, monoclonal gammopathy of undetermined significance; RPGN, rapidly progressive glomerulonephritis; AAV, ANCA-associated vasculitis; AKI, acute kidney injury; ARF, acute renal failure.

Figure S3

a

Age	Event (n/N)	Odds ratio (95% CI)
18–49	37 / 32 070	Reference
50–59	23 / 12 465	1.33 (0.78–2.25)
60–69	37 / 15 810	1.39 (0.87–2.24)
70–79	63 / 14 593	2.10 (1.35–3.26)
≥80	31 / 3 879	3.17 (1.89–5.34)

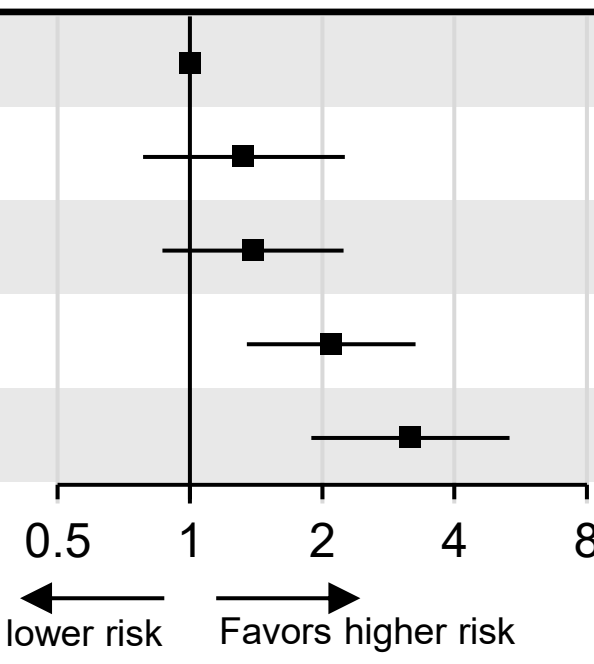

b

BMI	Event (n/N)	Odds ratio (95% CI)
<18.5	28 / 6 525	Reference
18.5–<22.0	63 / 23 958	0.69 (0.44–1.08)
22.0–<25.0	63 / 22 960	0.73 (0.46–1.15)
≥25.0	37 / 25 374	0.45 (0.27–0.75)

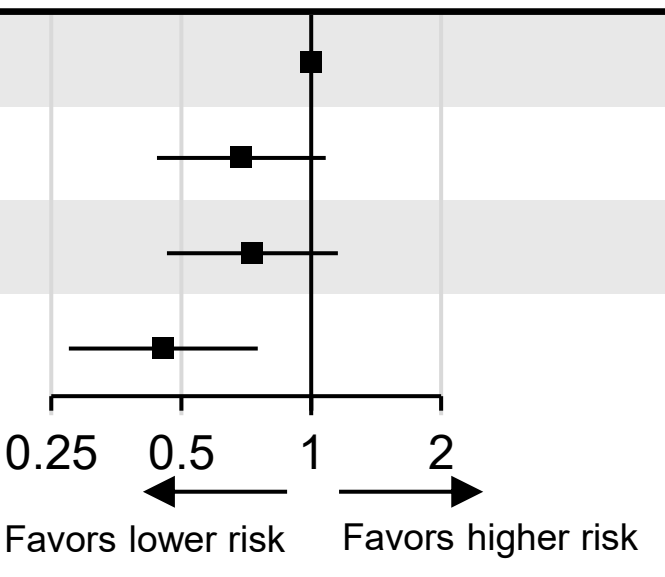

c

Prebiopsy diagnosis	Event (n/N)	Odds ratio (95% CI)
1. Chronic glomerulonephritis	31 / 36 295	Reference
2. Proteinuria / nephrotic syndrome	26 / 15 700	1.41 (0.83–2.41)
4. IgA vasculitis	3 / 1 535	2.15 (0.66–7.06)
7. Interstitial nephritis	4 / 1 786	1.80 (0.63–5.14)
10. Other kidney diseases	40 / 11 927	2.73 (1.68–4.45)
6. Other collagen diseases	6 / 1 068	4.00 (1.63–9.79)
5. SLE	1 / 786	2.67 (1.16–6.14)
9. MM, MGUS / amyloidosis	7 / 2 792	0.74 (0.10–5.52)
3. RPGN / AAV	50 / 5 422	6.42 (3.97–10.4)
8. AKI / ARF	23 / 1 506	10.8 (6.11–18.9)

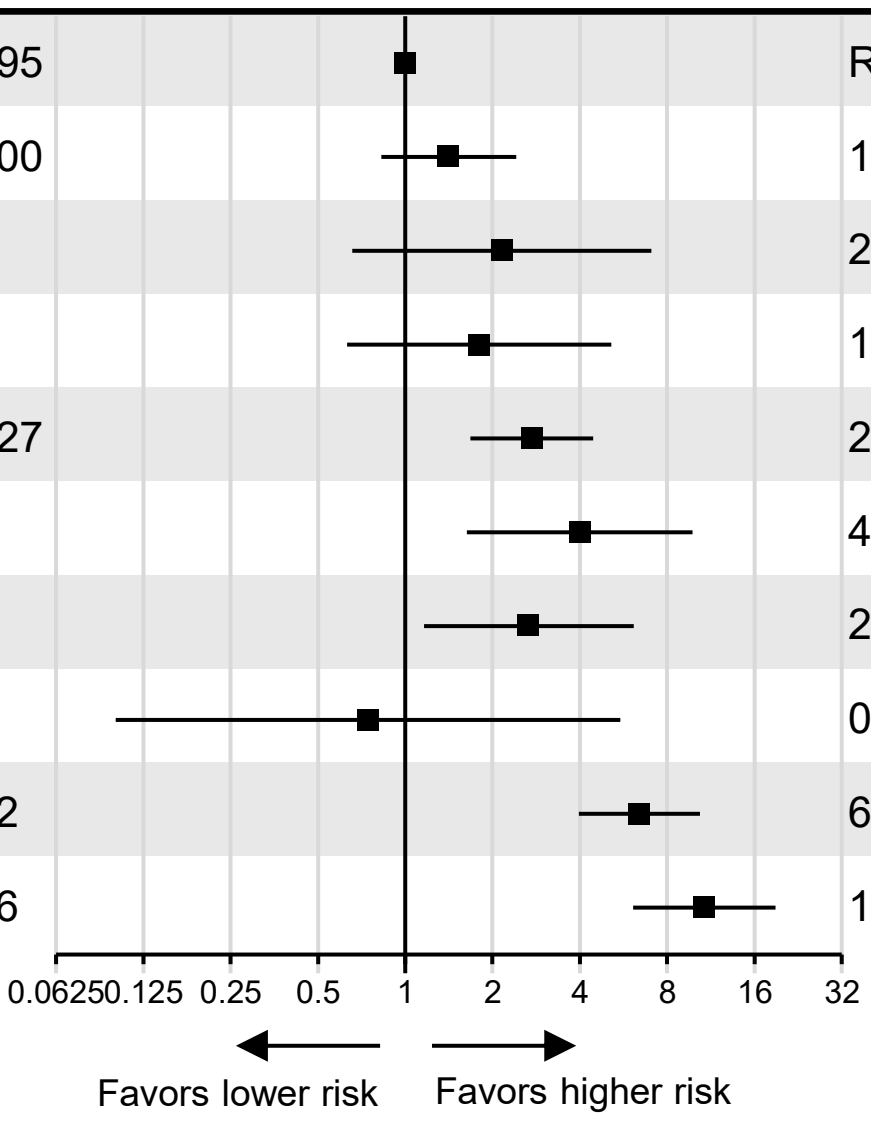

d

Hemostatic drug	Event (n/N)	Odds ratio (95% CI)
No	67 / 22 942	Reference
Tranexamic acid	5 / 3 677	0.52 (0.21–1.29)
Carbazochrome sulfonate	33 / 12 848	1.01 (0.66–1.54)
Combination therapy	86 / 39 350	0.82 (0.59–1.14)

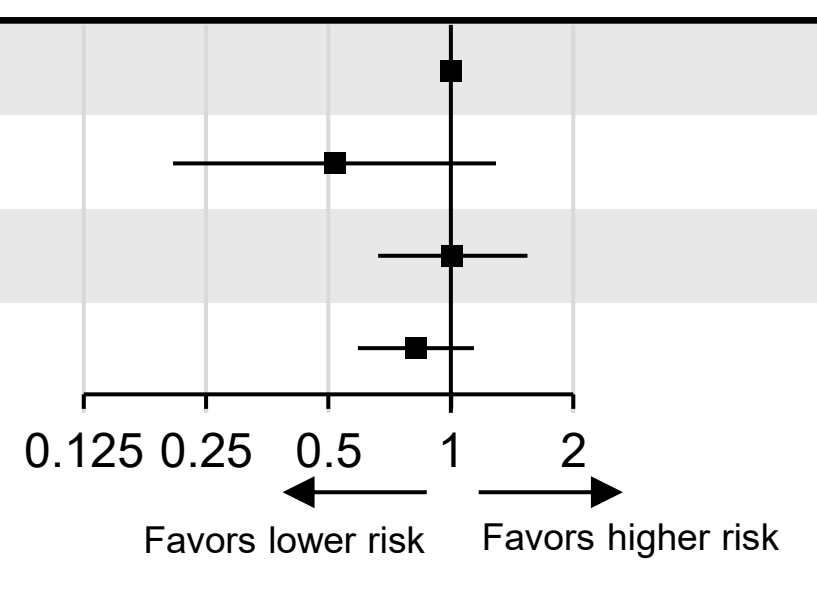

Figure S3. Adjusted odds ratios for ≥4-unit transfusion across clinical and treatment-related factors.

This figure presents forest plots showing adjusted odds ratios (ORs) and 95% confidence intervals (CIs) for the risk of receiving ≥4 units of red blood cell transfusion following percutaneous kidney biopsy. Estimates were derived from multivariable logistic regression models adjusted for biopsy year, age group, sex, body mass index (BMI), Charlson Comorbidity Index, diabetes, cardiovascular disease, and prebiopsy clinical diagnosis. **(a)** age-stratified estimates using the 18–49-year group as the reference. **(b)** BMI-stratified estimates with <18.5 kg/m² as the reference. **(c)** shows results across prebiopsy clinical diagnosis categories relative to chronic glomerulonephritis. **(d)** represents the association between haemostatic agent use and transfusion risk, with no haemostatic therapy as the reference category.

OR, odds ratio; CI, confidence interval; CGN, chronic glomerulonephritis; SLE, systemic lupus erythematosus; MM, multiple myeloma; MGUS, monoclonal gammopathy of undetermined significance; RPGN, rapidly progressive glomerulonephritis; AAV, ANCA-associated vasculitis; AKI, acute kidney injury; ARF, acute renal failure.

Figure S4

a Confusion matrix comparing actual bleeding outcomes with hypothetical hospitalization management assigned by a 95% cumulative probability threshold

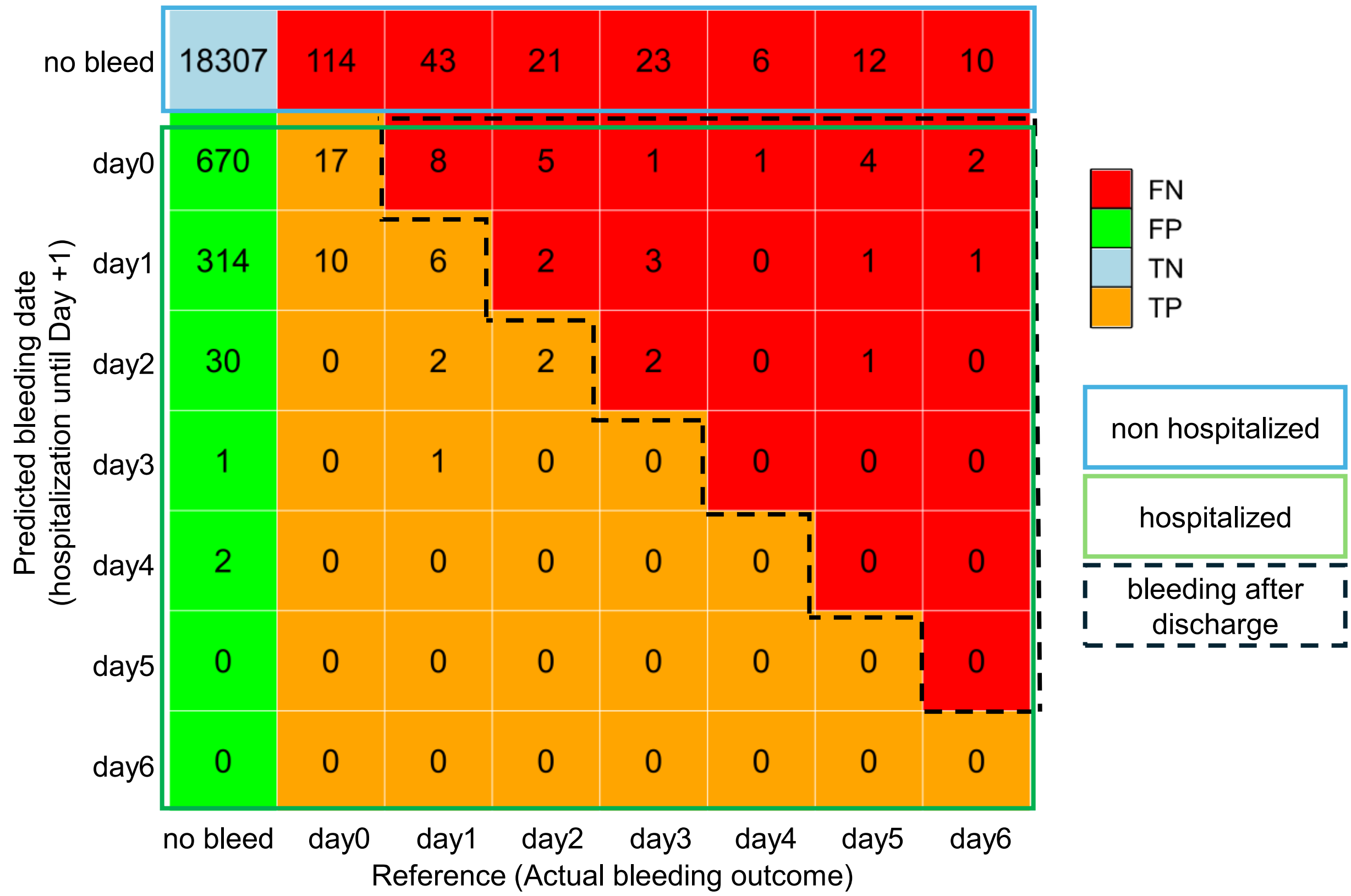

b Bleeding rates by predicted hospitalization status

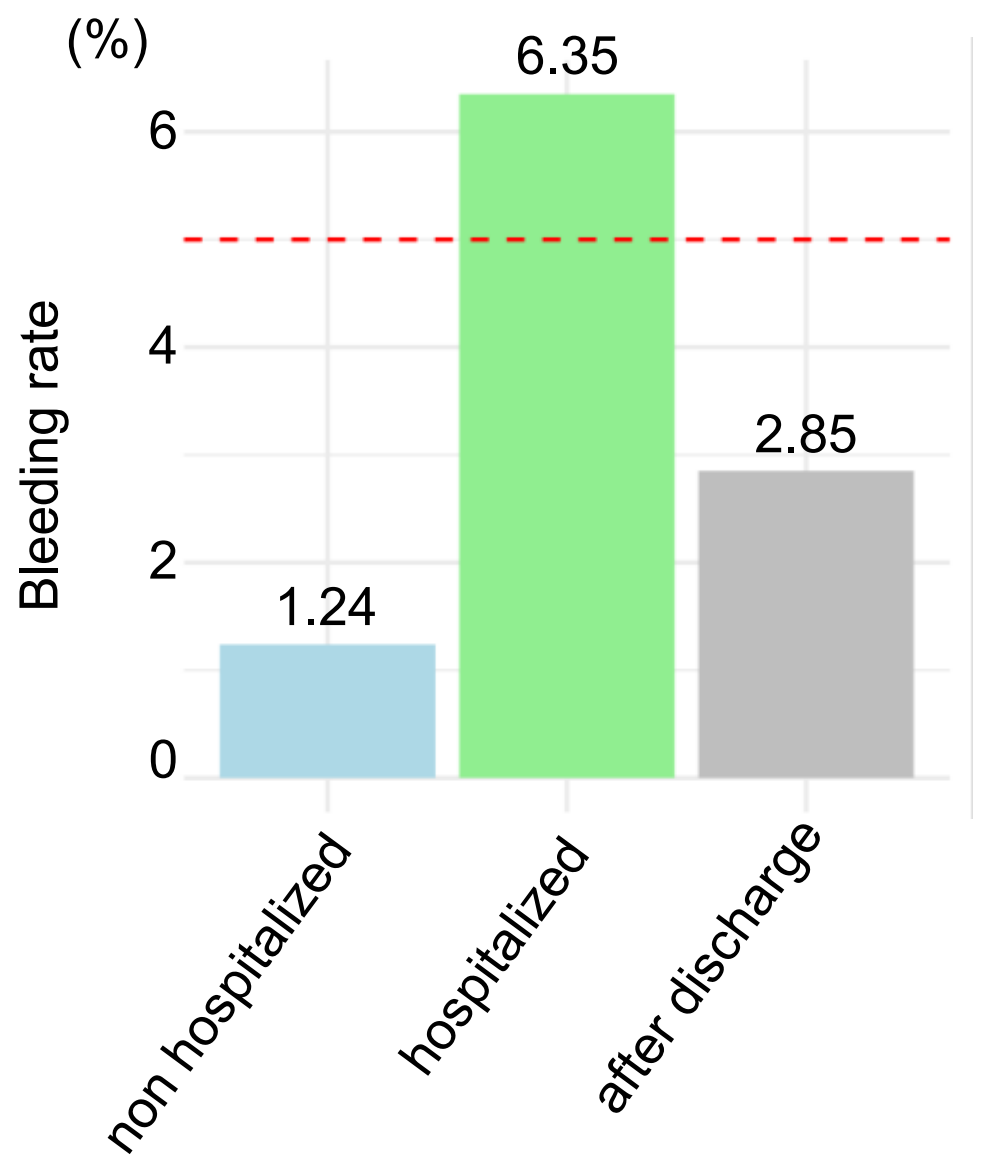

Figure S4. Descriptive visualization of model performance at a 0.95 threshold and bleeding rates across predicted outcome groups

(**a**) Confusion matrix showing the distribution of reference (actual bleeding outcome) versus predicted bleeding outcomes at a cumulative probability threshold of 0.95 and hospitalization decisions. Patients were assumed to remain hospitalized until the day after the predicted bleeding day. The cumulative probability threshold was applied solely for descriptive visualization and did not affect coefficient estimation. Bleeding events were classified as:

True Positive (TP): bleeding occurred within the predicted hospitalization period (orange)

False Positive (FP): no bleeding occurred during the predicted hospitalization period (green)

True Negative (TN): non-hospitalized and no bleeding occurred (gray)

False Negative (FN): bleeding occurred after the predicted hospitalization period or bleeding occurred without hospitalization (red)

For example, if bleeding occurred on Day 4 and the model predicted hospitalization until Day 5, this was counted as TP. The dashed line indicates bleeding after discharge.

(**b**) Bleeding rates by predicted hospitalization status: non-hospitalized (1.24%), hospitalized (6.35%), and bleeding after discharge (2.85%). The red dashed line represents the planned threshold of 5% for post-discharge bleeding risk. Highest bleeding risk is in the hospitalized group due to risk stratification.

STROBE Statement—Checklist of items that should be included in reports of ***cohort studies***

	Item No	Recommendation	Page/section where reported
**Title and abstract**	1	(*a*) Indicate the study's design with a commonly used term in the title or the abstract	Page 2
		(*b*) Provide in the abstract an informative and balanced summary of what was done and what was found	Page 3
**Introduction**			
Background/rationale	2	Explain the scientific background and rationale for the investigation being reported	Page 5
Objectives	3	State specific objectives, including any prespecified hypotheses	Page 6-7
**Methods**			
Study design	4	Present key elements of study design early in the paper	Page 7
Setting	5	Describe the setting, locations, and relevant dates, including periods of recruitment, exposure, follow-up, and data collection	Page 7
Participants	6	(*a*) Give the eligibility criteria, and the sources and methods of selection of participants. Describe methods of follow-up	Page 8
		(*b*) For matched studies, give matching criteria and number of exposed and unexposed	Not applicable
Variables	7	Clearly define all outcomes, exposures, predictors, potential confounders, and effect modifiers. Give diagnostic criteria, if applicable	Page 9-10
Data sources/ measurement	8*	For each variable of interest, give sources of data and details of methods of assessment (measurement). Describe comparability of assessment methods if there is more than one group	Page 8-9
Bias	9	Describe any efforts to address potential sources of bias	Page 7-11
Study size	10	Explain how the study size was arrived at	Not applicable
Quantitative variables	11	Explain how quantitative variables were handled in the analyses. If applicable, describe which groupings were chosen and why	Page 10
Statistical methods	12	(*a*) Describe all statistical methods, including those used to control for confounding	Page 9-11
		(*b*) Describe any methods used to examine subgroups and interactions	Supplementary file Page 2
		(*c*) Explain how missing data were addressed	Page 8
		(*d*) If applicable, explain how loss to follow-up was addressed	Not applicable
		(*e*) Describe any sensitivity analyses	Supplementary file Page 2
**Results**			
Participants	13*	(a) Report numbers of individuals at each stage of study—eg numbers potentially eligible, examined for eligibility, confirmed eligible, included in the study, completing follow-up, and analysed	Page 12
		(b) Give reasons for non-participation at each stage	Page 12
		(c) Consider use of a flow diagram	Figure 1
Descriptive data	14*	(a) Give characteristics of study participants (eg demographic, clinical, social) and information on exposures and potential confounders	Page 12

		(b) Indicate number of participants with missing data for each variable of interest	Figure 1
		(c) Summarise follow-up time (eg, average and total amount)	Page 12
Outcome data	15*	Report numbers of outcome events or summary measures over time	Page 19
Main results	16	(*a*) Give unadjusted estimates and, if applicable, confounder-adjusted estimates and their precision (eg, 95% confidence interval). Make clear which confounders were adjusted for and why they were included	Page 12‑13/ Table 2
		(*b*) Report category boundaries when continuous variables were categorized	Page 12-13
		(*c*) If relevant, consider translating estimates of relative risk into absolute risk for a meaningful time period	Not applicable
Other analyses	17	Report other analyses done—eg analyses of subgroups and interactions, and sensitivity analyses	Supplementary file Page 2
**Discussion**			
Key results	18	Summarise key results with reference to study objectives	Page 17
Limitations	19	Discuss limitations of the study, taking into account sources of potential bias or imprecision. Discuss both direction and magnitude of any potential bias	Page 20–21
Interpretation	20	Give a cautious overall interpretation of results considering objectives, limitations, multiplicity of analyses, results from similar studies, and other relevant evidence	Page 18‑21
Generalisability	21	Discuss the generalisability (external validity) of the study results	Page 21
**Other information**			
Funding	22	Give the source of funding and the role of the funders for the present study and, if applicable, for the original study on which the present article is based	Page 22

*Give information separately for exposed and unexposed groups.

Note: An Explanation and Elaboration article discusses each checklist item and gives methodological background and published examples of transparent reporting. The STROBE checklist is best used in conjunction with this article (freely available on the Web sites of PLoS Medicine at http://www.plosmedicine.org/, Annals of Internal Medicine at http://www.annals.org/, and Epidemiology at http://www.epidem.com/). Information on the STROBE Initiative is available at http://www.strobe-statement.org.

TRIPOD

TRIPOD Checklist: Prediction Model Development

Section/Topic	Item	Checklist Item	Page
**Title and abstract**			
Title	1	Identify the study as developing and/or validating a multivariable prediction model, the target population, and the outcome to be predicted.	Page 1
Abstract	2	Provide a summary of objectives, study design, setting, participants, sample size, predictors, outcome, statistical analysis, results, and conclusions.	Page 2
**Introduction**			
Background and objectives	3a	Explain the medical context (including whether diagnostic or prognostic) and rationale for developing or validating the multivariable prediction model, including references to existing models.	Page 5–6
	3b	Specify the objectives, including whether the study describes the development or validation of the model or both.	Page 6–7
**Methods**			
Source of data	4a	Describe the study design or source of data (e.g., randomized trial, cohort, or registry data), separately for the development and validation data sets, if applicable.	Page 7
	4b	Specify the key study dates, including start of accrual; end of accrual; and, if applicable, end of follow-up.	Page 7
Participants	5a	Specify key elements of the study setting (e.g., primary care, secondary care, general population) including number and location of centres.	Page 7
	5b	Describe eligibility criteria for participants.	Page 8
	5c	Give details of treatments received, if relevant.	NA
Outcome	6a	Clearly define the outcome that is predicted by the prediction model, including how and when assessed.	Page 9
	6b	Report any actions to blind assessment of the outcome to be predicted.	NA
Predictors	7a	Clearly define all predictors used in developing or validating the multivariable prediction model, including how and when they were measured.	Page 9–10
	7b	Report any actions to blind assessment of predictors for the outcome and other predictors.	NA
Sample size	8	Explain how the study size was arrived at.	NA
Missing data	9	Describe how missing data were handled (e.g., complete-case analysis, single imputation, multiple imputation) with details of any imputation method.	Page 8
Statistical analysis methods	10a	Describe how predictors were handled in the analyses.	Page 9–10
	10b	Specify type of model, all model-building procedures (including any predictor selection), and method for internal validation.	Page 9–10
	10d	Specify all measures used to assess model performance and, if relevant, to compare multiple models.	Page 14–16
Risk groups	11	Provide details on how risk groups were created, if done.	NA
**Results**			
Participants	13a	Describe the flow of participants through the study, including the number of participants with and without the outcome and, if applicable, a summary of the follow-up time. A diagram may be helpful.	Page 12
	13b	Describe the characteristics of the participants (basic demographics, clinical features, available predictors), including the number of participants with missing data for predictors and outcome.	Page 12
Model development	14a	Specify the number of participants and outcome events in each analysis.	Page 12
	14b	If done, report the unadjusted association between each candidate predictor and outcome.	Page 12–13
Model specification	15a	Present the full prediction model to allow predictions for individuals (i.e., all regression coefficients, and model intercept or baseline survival at a given time point).	Table2, S3

	15b	Explain how to the use the prediction model.	Page 15
Model performance	16	Report performance measures (with CIs) for the prediction model.	Page 16
**Discussion**			
Limitations	18	Discuss any limitations of the study (such as nonrepresentative sample, few events per predictor, missing data).	Page 20–21
Interpretation	19b	Give an overall interpretation of the results, considering objectives, limitations, and results from similar studies, and other relevant evidence.	Page 18
Implications	20	Discuss the potential clinical use of the model and implications for future research.	Page 21
**Other information**			
Supplementary information	21	Provide information about the availability of supplementary resources, such as study protocol, Web calculator, and data sets.	Page 22
Funding	22	Give the source of funding and the role of the funders for the present study.	Page 22

We recommend using the TRIPOD Checklist in conjunction with the TRIPOD Explanation and Elaboration document.
